# Set1-catalyzed H3K4 trimethylation antagonizes the HIR/Asf1/Rtt106 repressor complex to promote histone gene expression and chronological life span

**DOI:** 10.1093/nar/gkz101

**Published:** 2019-02-13

**Authors:** Qianyun Mei, Chen Xu, Madelaine Gogol, Jie Tang, Wanping Chen, Xilan Yu, Jerry L Workman, Shanshan Li

**Affiliations:** 1 State Key Laboratory of Biocatalysis and Enzyme Engineering, School of Life Sciences, Hubei University, Wuhan, Hubei 430062, China; 2 Stowers Institute for Medical Research, 1000 E. 50th Street, Kansas City, MO 64110, USA

## Abstract

Aging is the main risk factor for many prevalent diseases. However, the molecular mechanisms regulating aging at the cellular level are largely unknown. Using single cell yeast as a model organism, we found that reducing yeast histone proteins accelerates chronological aging and increasing histone supply extends chronological life span. We sought to identify pathways that regulate chronological life span by controlling intracellular histone levels. Thus, we screened the histone H3/H4 mutant library to uncover histone residues and posttranslational modifications that regulate histone gene expression. We discovered 15 substitution mutations with reduced histone proteins and 5 mutations with increased histone proteins. Among these mutations, we found Set1 complex-catalyzed H3K4me3 promotes histone gene transcription and maintains normal chronological life span. Unlike the canonical functions of H3K4me3 in gene expression, H3K4me3 facilitates histone gene transcription by acting as a boundary to restrict the spread of the repressive HIR/Asf1/Rtt106 complex from histone gene promoters. Collectively, our study identified a novel mechanism by which H3K4me3 antagonizes the HIR/Asf1/Rtt106 repressor complex to promote histone gene expression and extend chronological life span.

## INTRODUCTION

Aging is the leading risk factor for many human diseases, including cancer, diabetes, cardiovascular disorders, and neurodegenerative diseases (1). Given the fact that the molecules and fundamental mechanisms of aging are highly conserved across eukaryotes, studying aging in the unicellular yeast can shed light on the mechanisms relevant to mammalian aging (2). The budding yeast *Saccharomyces cerevisiae* has two different aging models: replicative life span and chronological life span. The replicative life span refers to the number of times a single yeast cell can divide and the chronological life span is the length of time that a post-mitotic cell survives. The chronological life span is a good model to study the aging related response and molecular changes within post-mitotic cells (3).

In eukaryotes, DNA is packaged into chromatin and its fundamental structural unit is the nucleosome. Two copies each of the core histone proteins H2A, H2B, H3 and H4 associate with DNA to form the histone octamer, which is wrapped by 147 bp DNA to form the nucleosome core (4). To maintain proper chromatin organization and function, cells need to not only synthesize a large number of histones but also restrict most histone synthesis to S phase. Insufficient histone levels may trigger a cell-cycle arrest in budding yeast and impair S phase progression in mammals (5,6). Reduced expression or depletion of core histones during DNA replication disrupts chromatin structure, delays S phase completion and results in mitotic arrest (5,7–9).

In budding yeast, there are two copies of four core histone genes. Each core histone gene is arranged in an opposite orientation to the gene encoding its interaction partner (10), i.e. *HHT1-HHF1* and *HHT2-HHF2* encode H3-H4 pairs; *HTA1-HTB1* and *HTA2-HTB2* encode H2A–H2B pairs. This divergent arrangement of histone genes allows coordinated expression to produce equal amount of four core histones. The intergenic regions of histone genes contain multiple copies of 16-bp UAS sequence (Upstream activating sequence). These UAS elements facilitate histone gene activation by recruiting transcription activators, including Spt10, Spt21, SBF and MBF (11). SBF is a heterodimer of Swi4 and Swi6 and MBF is composed of Mbp1 and Swi6 (12). Swi4 and Mbp1 are DNA-binding factors in SBF and MBF, respectively (12). The binding sites of Spt10 overlap and are mutually exclusive with those of SBF and MBF (13), implying that these transcription activators function in different pathways. In fact, SBF and Spt10 act together to control the timing of histone gene expression: SBF initiates a small early peak of histone transcription and Spt10 is responsible for the major late peak (13). The core histone gene pairs *HTA1-HTB1, HHT1-HHF1* and *HHT2-HHF2* also contain a specialized 54 bp negative regulatory element (NEG) to mediate the repression of histone gene expression (11). The NEG regions are bound by the histone regulation (HIR) complex composed of Hir1, Hir2, Hir3 and Hpc2, which then recruits Asf1, H3–H4 tetramers and Rtt106 (14). The chromatin remodeling complex (RSC) is recruited by Rtt106 and functions together with HIR/Asf1/Rtt106 to assemble repressive chromatin over histone gene promoters to occlude the basal transcription machinery (15,16). The repressive effect of Rtt106 is alleviated by Yta7, which functions as a boundary protein to limit the spread of Rtt106 to histone gene coding regions (14,17). However, during S phase, Yta7 is phosphorylated by cyclin-dependent kinase 1 and casein kinase 2 and needs to be released from histone gene coding regions to facilitate RNA polymerase II elongation (18). It remains unclear how the repressive HIR/Asf1/Rtt106 complex is restricted during S phase when histone genes are actively transcribed.

Yeast replicative aging is accompanied by reduced histone proteins, which is also a cause of aging in budding yeast (19). During replicative aging, nucleosome occupancy is decreased by 50% across the genome, leading to large-scale chromosomal alterations and transcriptional induction of most yeast genes (19). Aging-coupled histone loss also results in elevated levels of DNA strand breaks, mitochondrial DNA transfer to the nuclear genome, translocations, and retrotransposition (19). Increased histone supply can efficiently extend the replicative life span of budding yeast (20). However, little is known about chronological life span and histone protein levels. Here, we reported that reducing yeast histone proteins leads to accelerated chronological aging and increasing histone supply extends chronological life span. To identify chronological aging regulators, we screened the yeast histone H3/H4 mutant library for histone residues and posttranslational modifications (PTMs) that regulate histone protein levels. We found that Set1-catalyzed H3K4me3 facilitates histone gene transcription and maintains normal chronological life span. Set1-catalyzed H3K4me3 promotes histone gene expression by antagonizing the repressive effect of HIR/Asf1/Rtt106 complex. Our study provides novel insights into histone gene regulation as well as life span regulation.

## MATERIALS AND METHODS

### Materials

Histone H3 and H4 Mutant Collection (YSC5105, YSC5106) was purchased from GE Healthcare. All yeast strains used in this study are described in Supplementary Table S1. Primers for qPCR are listed in Supplementary Table S2. All antibodies and other critical reagents used in this study are described in Supplementary Table S3.

### Histone extraction, preparation of yeast whole cell extracts

Histones were extracted from exponential growing yeast cells as described previously (21). Cells were grown in 1–5 ml YPD (Yeast Extract Peptone Dextrose) or selective medium as indicated until OD_600_ of 0.7–1.0. Cells were then harvested and lysed in 2 M NaOH with 8% β-mercaptoethanol. Cell lysate was centrifuged and the pellet was washed twice with TAP extraction buffer (40 mM HEPES–KOH pH7.5, 10% glycerol, 350 mM NaCl, 0.1% Tween-20). Cell pellets were resuspended in 2× SDS-sample buffer. For immunoprecipitation, 200 ml cells were grown in YPD until OD_600_ of 1.0, harvested and lysed with glass beads followed by centrifugation as described previously (21).

### Western blots analysis

Protein samples were separated by 8–15% SDS-PAGE and transferred to Immobilon-P PVDF membrane. The blots were probed with antibodies against specific proteins followed by incubation with horseradish peroxidase-labeled IgG secondary antibodies. The specific proteins were visualized by using the ECL Chemiluminescence Detection Kit. Western blots were quantified with Image J software.

For quantitative Western blots analysis with Li-Cor Odyssey, IRDye 680RD goat anti-mouse and IRDye 800CW goat anti-rabbit secondary antibodies were used. Membranes were scanned with a Li-Cor Odyssey infrared imaging system, and the fluorescence intensity was quantitated using the associated Odyssey software (20).

### Fractionation of soluble histones from histones in chromatin

Chromatin fractionation was performed as described previously with modifications (20). 50 ml exponential growing cells were spheroplasted in SB (1 M sorbitol, 20 mM Tris pH 7.4, 10 mg/ml zymolyase 20T) and lysed with EBX (20 mM Tris pH 7.4, 100 mM NaCl, 0.5% Triton X-100, 15 mM β-ME + protease inhibitors). The lysate was centrifuged over NIB (20 mM Tris pH 7.4, 100 mM NaCl, 1.2M sucrose, 15 mM β-ME + protease inhibitors). The nuclear pellet was then lysed with 1% Triton X-100 and centrifuged. Proteins in the supernatant (soluble fractions) and the pellet (chromatin fractions) were resuspended in SDS-PAGE sample buffer.

### Quantitative reverse transcription PCR

RNA was isolated from exponential growing yeast cells by standard phenol-chloroform extraction procedures. Purified RNA was then subjected to quantitative reverse transcription PCR (qRT-PCR) as described previously (21,22). Results were analyzed using ΔΔCt.

### Genomic DNA isolation and quantification

To quantitate mRNA level to genomic DNA (gDNA) in Figure 5A and D, exponential growing yeast cells were divided into two aliquots, one part for mRNA extraction and the other for gDNA extraction. RNA was isolated by standard phenol-chloroform extraction procedures and gDNA was isolated as described previously (20). DNA was resuspended in TE and DNA concentrations were determined using a NanoDrop spectrophotometer. Proper diluted gDNA and mRNA-derived cDNA were used in qPCR with specific primers. Results were presented as relative RNA levels normalized to their gDNA levels.

### Chromatin immunoprecipitation (ChIP) assay

The ChIP assays were performed as previously described (21). Yeast cells were grown in 200 ml YPD media at 28°C until OD_600_ of 0.5–0.7. Crosslinking was performed by adding 5.6 ml 37% formaldehyde to a final concentration of 1% and quenched by adding 10 ml of 2.5 M glycine. Cells were then harvested, washed and lysed with FA-SDS lysis buffer (0.1% SDS, 40 mM HEPES–KOH, pH7.5, 1 mM EDTA pH 8.0, 1% Triton X-100, 0.1% Na deoxycholate, 1 mM PMSF, 2 μg/ml leupeptin, 1 μg/ml pepstatin A, protease inhibitor cocktail). DNA was sheared by sonication and subjected to immunoprecipitation with antibodies pre-bound to Protein G Dynabeads. Beads were washed sequentially with FA lysis buffer, FA buffer with 500 mM NaCl, TEL buffer (10 mM Tris pH 8.0, 1 mM EDTA, 0.25 M LiCl, 1% NP-40, 1% Na deoxycholate) and TE (10 mM Tris pH 7.4, 1 mM EDTA). The eluted DNA/protein complexes were treated with 20 μg Proteinase K at 55°C for 1 h and reverse crosslink at 65°C overnight. Purified RNase A digested DNA were quantitated by qPCR.

For ChIP-seq, library was constructed, sequenced and analyzed as described previously (21). Data was aligned to yeast genome sacCer3 from UCSC using bowtie2 version 2.1.0 with parameter -k 1. Data was read into R (3.1.0) for further analysis. Peaks were called using a custom perl script, requiring a peak to have a minimum width of 50 bases and a threshold of 1.5-fold in IP versus input. Peaks that were within 400 bases of each other were merged. Bedtools 2.19.0 was used to annotate closest gene for each peak. Gene annotations used were from Ensembl 72. There are three biological replicates for H3K4me3/H3 ChIP.

### Measurement of chronological life span

The yeast chronological life span was determined as described previously (2). A total of 5 ml of seed cultures from individual colonies were grown overnight in SDC medium (synthetic complete (SC) medium with 2% glucose). To examine the effect of overexpression of histone H3 and H4 on chronological life span, we grew cells in SC-leucine (SC-Leu) + 2% galactose. Flasks with 50 ml of appropriate media were seeded to generate an initial OD_600_ of 0.1 and incubated for 3 days until cell growth was ceased. On day 3, 100 μl aliquots of each culture were collected and 10-fold serial dilution was created and spread on YPD plate to determine the colony-forming units (CFU). At subsequent time points, additional 100 μl aliquots were removed from the still-shaking 50 ml cultures, diluted, plated and counted. The CFU score for each culture decreased as a function of time, generating a life span curve.

### Immunoprecipitation

Immunoprecipitation was performed as described previously (21). Yeast whole cell extract was digested with Micrococcal Nuclease and then mixed with 30 μl anti-FLAG M2 agarose for 1 h at 4°C in IP binding buffer (40 mM HEPES–KOH, pH7.5, 0.1% NP-40, 10% glycerol, 1 mM PMSF, 150 mM NaCl, 2 μg/ml leupeptin, 1 μg/ml pepstatin A, protease inhibitor cocktail). The beads were washed three times with a large excess of IP washing buffer (40 mM HEPES–KOH, pH 7.5, 0.1% NP-40, 10% glycerol, 1 mM PMSF, 350 mM NaCl, 2 μg/ml leupeptin, 1 μg/ml pepstatin A). Supernatants from the boiled beads were subjected to SDS PAGE and western blots. For *in vitro* immunoprecipitation, Rtt106-FLAG was purified by anti-FLAG M2 affinity gel as described previously (21). Purified Rtt106 was then incubated with 0.2 μg recombinant purified histones for 0.5 h at 4°C, immunoprecipitated with anti-FLAG M2 agarose and washed three times with a large excess of IP washing buffer. Supernatants from the boiled beads were subject to SDS-PAGE and Western blots. For *in vitro* immunoprecipitation with purified recombinant octamers, purified Rtt106 was incubated with 2.4 μg recombinant purified octamers for 1 h at 4°C, immunoprecipitated with anti-FLAG M2 agarose and then washed three times with a large excess of IP washing buffer.

### Statistical analysis

Statistical differences in this study were determined by two-tailed unpaired *t*-test and a *P*-value <0.05 was considered statistically significant.

## RESULTS

### Intracellular histone protein levels regulate chronological life span in *Saccharomyces cerevisiae*

**Figure 1.**
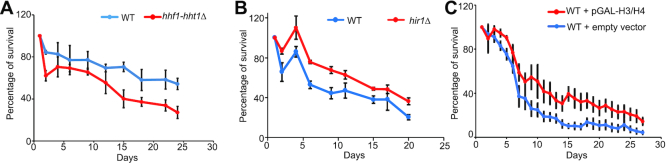
Histone levels regulate chronological life span. (**A**) Analysis of chronological life span of WT and *hhf1-hht1*Δ mutant in SDC medium. During chronological aging, the survival percentage of WT and *hhf1-hht1*Δ mutant is determined by their ability to form colonies on YPD plates. (**B**) Analysis of chronological life span of WT and *hir1*Δ mutant in SDC medium. (**C**) Ectopic expression of histone proteins from a divergent galactose-inducible promoter extends the chronological life span. WT cells transformed with pGAL-H3/H4 or empty vector were grown in SC-Leu+2% galactose to induce the ectopic expression of histones.

A profound loss of histone proteins was observed during yeast replicative aging and this loss of histones is a causal factor in replicative aging (20). However, it remains unclear if there is a causal connection between histone protein levels and chronological life span. To examine the effect of histone loss on yeast chronological life span, we deleted *HHF1-HHT1* (*hhf1-hht1Δ, HHT1* encodes H3 and *HHF1* encodes H4) to reduce the expression of histones (Supplementary Figure S1A). Using the chronological life span assay, we found that *hhf1-hht1Δ* mutant has shortened life span compared with its wild-type strain (Figure 1A), indicating that similar to the situation in replicative life span, reducing histone protein levels accelerates chronological aging. Next, we examined the effect of increasing histones supply on yeast chronological life span. Histone gene transcription is repressed by HIR H3/H4 histone–protein chaperone complex (Hir1, Hir2, Hir3 and Hpc2) (23,24). Expression of histone H3 was increased in *hir1Δ, hir2Δ*, and *hir3Δ* mutants (Supplementary Figure S1B). We found that *hir1Δ* mutant has extended chronological life span when compared to its wild-type cells (Figure 1B). Moreover, we examined the effect of overexpression of histone H3 and H4 from a galactose-inducible promoter (pGAL-H3/H4) on yeast chronological life span. Cells transformed with the pGAL-H3/H4 plasmid have increased intracellular histones and extended chronological life span compared with cells transformed with the control empty vector when grown in galactose-containing medium (Figure 1C, Supplementary Figure S1C), supporting the concept that increasing the supply of histone proteins can efficiently extend the longevity of budding yeast. Collectively, these data indicate that it is crucial to maintain intracellular histone proteins to prevent abnormal chronological aging.

### Screen for histone residues that regulate histone gene expression

**Figure 2.**
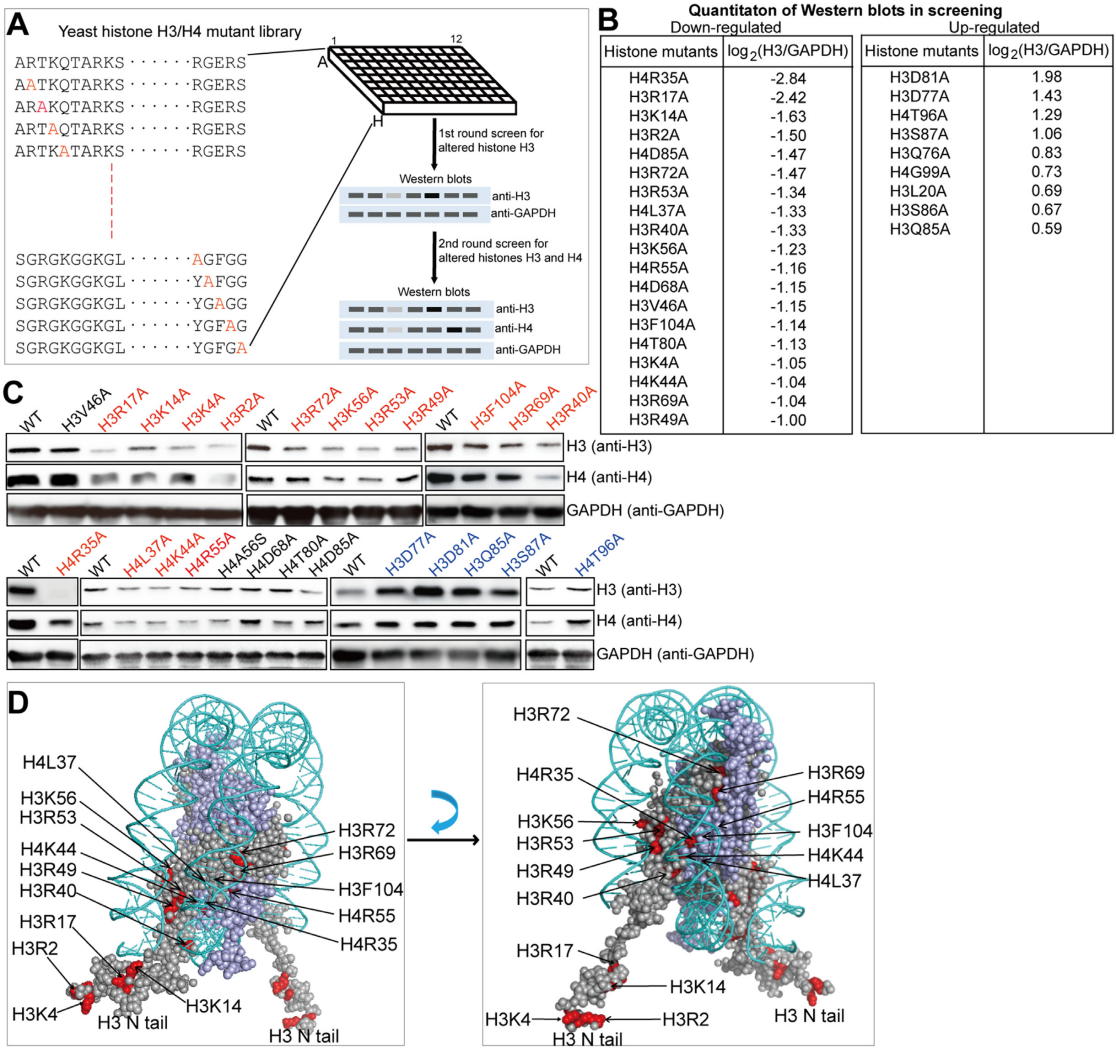
Screen for histone residues that regulate histone gene expression. (**A**) Diagram for experimental design. 196 substitution mutants from histone H3/H4 mutant library were grown in YPD medium until OD_600_ of 0.7–1.0. Cells were harvested and proteins were extracted. The first round of screen was to identify histone mutants that have altered histone H3 proteins. For histone mutants that have altered histone H3 levels, the second round of screen was performed to further confirm their effects on histone proteins by examining both H3 and H4 levels. (**B**) Quantitation results of the first round of mutational screen for regulators of histone H3. All Western blots from the first round of screen were quantitated by Image J with a form of log2(H3/GAPDH). Shown here are the mutations with at least 2-fold reduced H3 (log2(H3/GAPDH) ≤ –1) and the mutations with more than 1.5-fold increased H3 (log2(H3/GAPDH) ≥ 0.58). (**C**) The second round of screen for histone mutants that have similar changes of histones H3 and H4. The mutations having reduced histones H3 and H4 were highlighted with red and increased histones H3 and H4 were highlighted with blue. (**D**) Representation of the nucleosome, highlighting the residues essential for maintaining histone proteins (in red color). PDB file 1KX5 was used.

Next, we aimed to identify factors that regulate chronological life span by modulating intracellular histone protein levels, especially with regards to histone gene regulation. In general, gene transcription is tightly linked to histone posttranslational modifications (PTMs) and several histone PTMs have been reported to regulate histone gene expression, such as H3K56 acetylation (H3K56ac) and H2B tyrosine 37 phosphorylation (H2BY37) (25,26). Despite this progress, little is known about the effect of most histone PTMs on histone gene expression. Thus, we sought to identify potential histone PTMs that regulate histone proteins by analyzing the global levels of histones in strains from a yeast histone H3/H4 mutant library by Western blots. This library contains individual substitution mutations for all residues of histone H3 and H4, except for 32 inviable mutations (27). We first screened 196 substitution mutants for altered histone H3 levels when grown to mid-log phase (Figure 2A). Our initial screening identified a total of 28 substitutions with dramatic altered histone H3 changes: 19 substitutions with 2-fold reduced histone H3 and H9 substitutions with 1.5-fold increased histones (Figure 2B, Supplementary Figure S2). For further confirmation, another round of screen was performed by examining the levels of both histone H3 and H4 in these 28 mutants (Figure 2A). Our data showed that the protein levels of H3 and H4 were significantly reduced in 15 histone substitution mutants and increased in 5 histone substitution mutants (Figure 2C). Interestingly, 13 out of 15 substitutions with reduced histones occurred on arginine (R) or lysine (K) residues (Figure 2C). Eleven substitutions with reduced histones occurred on H3 including four residues in the N-terminal tails (H3R2A, H3K4A, H3K14A, H3R17A) and seven residues within the globular domain (H3R40A, H3R49A, H3R53A, H3K56A, H3R69A, H3R72A, H3F104A) (Figure 2D). Four substitutions with reduced histones occurred on H4 (H4R35A, H4L37A, H4K44A, H4R55A) (Figure 2D). The five substitutions that have increased histone proteins are H3D77A, H3D81A, H3Q85A, H3S87A and H4T96A (Figure 2C).

Among these residues, H3R2, H3K4, H3K14 and H3K56 have been reported to undergo posttranslational modifications in budding yeast (28). The reduced histone proteins in the H3K56A mutant is consistent with a previous study showing that H3K56 acetylation (H3K56ac) regulates histone gene expression (25). H3K4 can be trimethylated (H3K4me3) and H3R2A and H3K14A have been reported to regulate H3K4me3 (29,30). Most importantly, H3K4 methylation has been shown to play critical roles in gene expression and is highly conserved from yeast to mammals (31); we therefore focused on H3K4 in the following studies.

### Set1-catalyzed H3K4me3 is required to maintain normal histone protein levels

**Figure 3.**
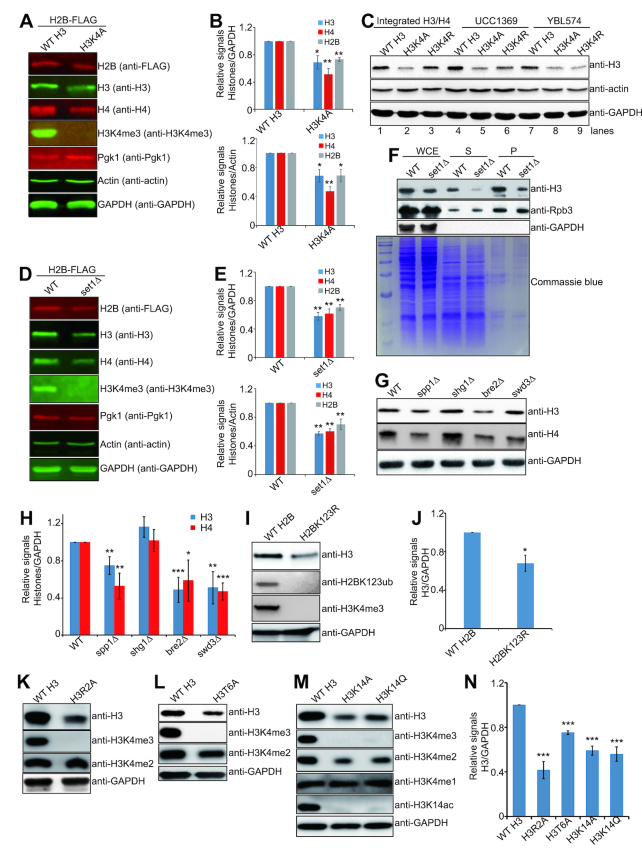
Set1-catalyzed H3K4me3 is required to maintain normal histone protein levels. (**A** and **B**) Western blots analysis of histone proteins in exponential growing yeast cells (H2B-FLAG WT H3, H2B-FLAG H3K4A). The relative intensities of protein levels were determined by quantitative Western blots analysis using a Li-Cor Odyssey system. Histones were normalized to GAPDH and Actin, separately. (**C**) Western blots analysis of histone proteins in WT H3 and H3K4 mutants (H3K4A, H3K4R) in different strain backgrounds (Integrated H3/H4, UCC1369, YBL574). (**D** and **E**) Western blots analysis of histone proteins in exponential growing yeast cells (H2B-FLAG WT, H2B-FLAG *set1Δ*). The relative intensities of protein levels were determined by quantitative Western blots analysis using a Li-Cor Odyssey system. (**F**) Effects of Set1 on nonchromatin-bound histones (soluble nucleus fraction, S) and chromatinized histones (P) by Western blots. Total (WCE), soluble (S) and pellet (P) proteins were extracted from exponential growing cells (WT, *set1Δ*). Below is a Coomassie-stained gel of the same amounts of the same total yeast protein extracts analyzed in the Western blots analysis. RNA polymerase II subunit Rpb3 was used as a nuclear loading control. (**G** and **H**) Western blots analysis of histone proteins in exponential growing yeast cells (WT, *spp1Δ, shg1Δ, bre2Δ, swd3Δ*). The relative intensities of Histones/ GAPDH were quantified using Image J with standard error (SE). (**I** and **J**) Western blots analysis of histone proteins in exponential growing yeast cells (WT H2B, H2BK123R). (K-N) Western blots analysis of histone proteins in exponential growing yeast cells (WT H3, H3R2A, H3T6A, H3K14A, H3K14Q). The relative intensities of Histone H3/GAPDH were quantified using Image J with standard error (SE). Data represent the mean ± SE of three independent experiments. (*) *P* < 0.05; (**) *P* < 0.01; (***) *P* < 0.001, unpaired *t*-test two-tailed *P*-value compared with the corresponding wild type.

We found that the H3K4A substitution had significantly reduced histones H2B and H4 in addition to histone H3 (Figure 3A and B, Supplementary Figure S3A and B). Histone proteins were also found to be significantly reduced in H3K4Q, H3K4R and H3K4M mutants (Supplementary Figure S3C, D, E and F). To exclude the possibility that the above observed reduced histone proteins in H3K4 mutants were caused by yeast genetic backgrounds, we examined the effect of H3K4 mutation on histone proteins in three different strain backgrounds. The first two strains are histone shuffle strains, YBL574 (21) and UCC1369 (32), which harbor a *HHF1-HHT1* containing plasmid that encodes wild-type histone H3 and H4. We constructed YBL574 (H3K4A, H3K4R) and UCC1369 (H3K4A, H3K4R) mutants by individually transforming plasmids containing H3K4A or H3K4R into YBL574 and UCC1369 and selecting on 5-fluoroorotic acid (5-FOA) or α-aminoadipic acid to remove the wild-type histone plasmid containing *URA3* or *LYS2* gene. In exponential growing YBL574 (H3K4A, H3K4R) and UCC1369 (H3K4A, H3K4R) mutants, we observed remarkably reduced histones when compared to their wild-type counterparts (Figure 3C, lanes 4–6 and lanes 7–9). To exclude the effects of plasmids on histone proteins, wild-type H3 (WT H3) or H3K4 mutations (H3K4A, H3K4R) were integrated into yeast genome and histones were found to be remarkably reduced in both H3K4A and H3K4R mutants (Figure 3C, lanes 1–3). Collectively, these data indicate that mutation of H3K4 significantly reduced histone protein levels.

The reduced histone protein levels in the H3K4 mutants could be caused by H3K4 posttranslational modifications that regulate histone gene expression. As H3K4 methylation is solely catalyzed by the histone methyltransferase Set1 in budding yeast, it is much easier to investigate the effect of H3K4 methylation on histone protein levels. Deletion of *SET1* completely abolished H3K4 methylation and significantly reduced global levels of histones H2B, H3 and H4 (Figure 3D and E, Supplementary Figure S3G and H). In contrast, histone H3 was not significantly reduced in *set2Δ* mutant, which abolished H3K36 methylation (Supplementary Figure S3I and J), indicating that H3K4 methylation is specifically required to maintain normal histone protein levels. We also compared the effect of *SET1* deletion on chromatinized histones and unassembled free histones. Chromatin and soluble nuclear fractions were isolated by chromatin fractionation from exponential growing WT and *set1Δ* cells. Histone proteins in both chromatin and soluble nuclear fractions were reduced in *set1Δ* mutant (Figure 3F), indicating that Set1 regulates both chromatinized and unassembled free histone protein levels.

To identify whether H3K4 monomethylation (H3K4me1), H3K4 dimethylation (H3K4me2), or H3K4 trimethylation (H3K4me3) is required to maintain normal histone proteins, we analyzed histone proteins in Set1 complex subunit mutants that differentially impair H3K4 methylation status. Among the Set1 complex subunits, Bre2, Swd2, and Swd3 are required for H3K4me1/me2/me3; Spp1 is required for H3K4me3, while loss of Shg1 has no effect on H3K4 methylation (21). H3K4me1/me2/me3 and histones H3 and H4 were significantly reduced in *bre2Δ* and *swd3Δ* mutants but not in *shg1Δ* mutant (Figure 3G and H, Supplementary Figure S3K), confirming the requirement of H3K4 methylation to maintain normal histone protein levels. Moreover, we observed reduced H3K4me3 as well as reduced histone protein levels in *spp1Δ* mutant (Figure 3G and H, Supplementary Figure S3L and M), indicating that H3K4me3 is required for maintenance of intracellular histone proteins.

Set1-catalyzed H3K4me3 is regulated by other histone posttranslational modifications. To further confirm the role of H3K4me3 in regulating histone H3 protein levels, we examined the effect of histone mutations that specifically regulate H3K4me3 on histone H3 protein levels. Rad6/Bre1-catalyzed monoubiquitination of H2B at K123 (H2BK123ub) has been reported to be required for H3K4me3 (33). We thus analyzed histone proteins in H2BK123R mutant, which has no H2BK123ub as well as H3K4me3 (Figure 3I). Histone H3 was significantly reduced in H2BK123R mutant (Figure 3I and J). We also observed significantly reduced histone proteins in *bre1Δ* and *rad6Δ* mutants, neither of which have H2BK123ub nor H3K4me3 (Supplementary Figure S3N and O). Moreover, we examined histone protein levels in *UBP8* and *UBP10* deletion mutants, which encode enzymes that remove H2BK123ub (34). As expected, histone H3 was significantly increased in *ubp8Δ* and *ubp10Δ* mutants (Supplementary Figure S3P and Q). H3K4me3 has been reported to be regulated by H3R2 asymmetric dimethylation (H3R2me2a) and H3K4me3 was abolished in H3R2A mutant (Figure 3K) (29). Histone proteins were significantly reduced in H3R2A mutant (Figure 3K and N), consistent with our screening results (Figure 2B, Supplementary Figure S2). Histone proteins were also significantly reduced in H3T6A (Figure 3L and N) and H3K14 mutants (H3K14A and H3K14Q) (Figure 3M and N), which have been shown to eliminate H3K4me3 but have no effect on H3K4me1/2 (30,35). Collectively, these data indicate that Set1-catalyzed H3K4me3 is required to maintain normal histone protein levels.

### Set1-catalyzed H3K4me3 regulates chronological life span by promoting histone gene expression

**Figure 4.**
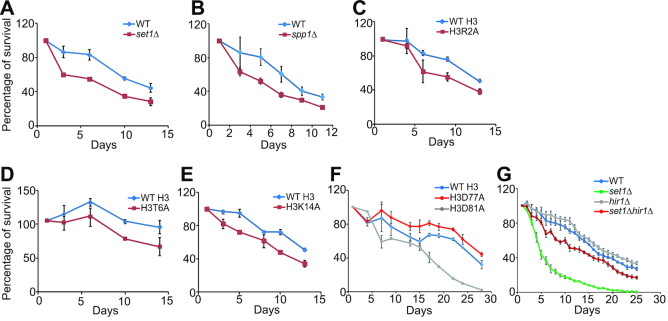
Set1-catalyzed H3K4me3 is required for normal chronological life span by promoting histone gene expression. Analysis of chronological life span of WT, *set1Δ, spp1Δ, hir1Δ, set1Δhir1Δ*, WT H3, H3R2A, H3T6A, H3K14A, H3D77A and H3D81A mutants. All data shown are mean ± SE of three independent experiments.

We examined the effect of Set1-catalyzed H3K4me3 on chronological life span of yeast cells. Similar to *hhf1-hht1Δ* mutant, deletion of *SET1* or *SPP1* reduced chronological life span (Figure 4A and B), indicating that Set1 complex-catalyzed H3K4me3 plays an important role in protecting cells from premature chronological aging. Similar to *set1Δ* and *spp1Δ* mutants, the chronological life span of H3R2A, H3T6A and H3K14A mutants was also significantly reduced (Figure 4C–E), supporting that the loss of H3K4me3 and reduced histone gene expression accelerate cell aging. In contrast, for H3D77A mutant, which has significant more histones than WT H3 (Supplementary Figure S4), it has extended life span compared with WT H3 (Figure 4F), further supporting our conclusion that increasing the supply of histone proteins can efficiently extend the longevity. We also noted that H3D81A mutant, which has much more histones (>2-fold) than H3D77A mutant (Supplementary Figure S4), has shortened chronological life span (Figure 4F), indicating that while modest histone increases are beneficial to life span, excessive accumulation of histones is not beneficial for normal chorological life span.

To show that Set1-catalyzed H3K4me3 regulates chronological life span by controlling histone proteins, we examined whether increasing histone supply by deleting *HIR1* could extend the chronological life span of short-lived *set1Δ* mutant. By comparing the chronological life span of WT, *set1Δ, hir1Δ* and *set1Δhir1Δ* mutants, we found that *set1Δhir1Δ* has an extended life span compared with that of *set1Δ* (Figure 4G). Together, these data indicate that Set1-catalyzed H3K4me3 extends chronological life span by promoting histone gene expression.

### Set1-catalyzed H3K4me3 positively regulates histone gene transcription

The reduced histone protein levels in *set1Δ* or H3K4 mutants could be caused by instability of histone proteins. We thus analyzed histone protein levels in the presence of the proteasome inhibitor, MG132 in WT H3 and H3K4A mutant. Histone H3 was reduced in H3K4A mutant even in the presence of MG132 (Supplementary Figure S5A), indicating that Set1-catalyzed H3K4me3 does not affect the stability of histone proteins.

**Figure 5.**
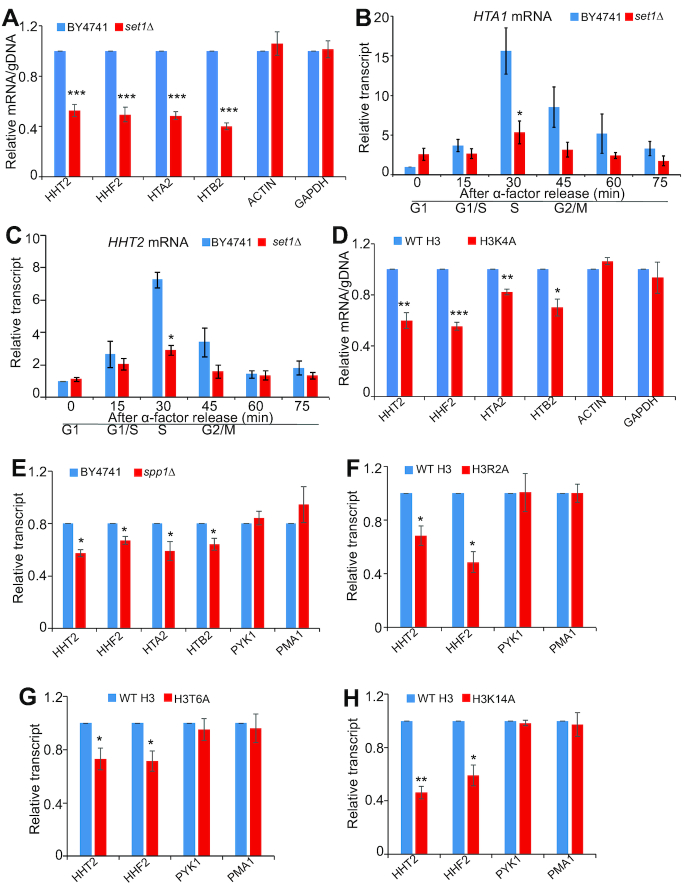
Set1-catalyzed H3K4me3 regulates histone gene transcription. (**A**) RNA levels for *HHT2, HHF2, HTA2, HTB2, ACTIN*, and *GAPDH* in exponential growing yeast cells (WT, *set1Δ*). The RNA levels of these genes were normalized to their DNA contents. Data represent the mean ± SE of three independent experiments. (B and C) qRT-PCR analysis of the transcription of *HTA1* (**B**) and *HHT2* (**C**) in α factor-synchronized WT and *set1Δ* mutant. Cells were arrested in G1 phase with α factor and then released into fresh medium, and samples were then taken at the indicated time points. The RNA levels of these genes were normalized to *ACTIN*. (**D**) RNA levels for *HHT2, HHF2, HTA2, HTB2, ACTIN*, and *GAPDH* in exponential growing yeast cells (WT H3, H3K4A). The RNA levels of these genes were normalized to their DNA contents. Data represent the mean ± SE of three independent experiments. (**E**) qRT-PCR analysis of the transcription of *HHT2, HHF2, HTA2* and *HTB2* in exponential growing yeast cells (WT, *spp1Δ*). *PYK1* and *PMA1* were used as negative controls. The RNA levels of these genes were normalized to *ACTIN*. (F–H) qRT-PCR analysis of the transcription of *HHT2, HHF2, PYK1* and *PMA1* in exponential growing yeast cells (WT H3, H3R2A (**F**), H3T6A (**G**), H3K14A (**H**)). The RNA levels of these genes were normalized to *ACTIN*. Data represent the mean ± SE of three independent experiments. (*) *P* < 0.05; (**) *P* < 0.01; (***) *P* < 0.001, unpaired *t*-test two-tailed *P*-value compared with the corresponding wild type.

Next, we examined the effect of Set1-catalyzed H3K4me3 on histone gene expression. By analyzing the transcriptome data for WT and *set1Δ* mutant (36), we found that the expression of canonical histone genes including *HTA1, HTB1, HTA2, HTB2, HHF2* and *HHT2* were reduced in *set1Δ* mutant (Supplementary Figure S5B). To confirm that, we analyzed histone transcripts in WT and *set1Δ* mutant by quantitative reverse transcription PCR (qRT-PCR) and found that the transcription of histone genes *HHT2, HHF2, HTA2* and *HTB2* was significantly reduced in *set1Δ* mutant when their transcripts were normalized to *ACTIN* (Supplementary Figure 5C). We also normalized histone transcripts to their genomic DNA (gDNA) contents (20) and found that the transcripts of histone genes were significantly reduced in *set1Δ* mutant while the transcripts of *ACTIN* and *GAPDH* remained unchanged (Figure 5A). We also investigated the effect of Set1 on histone gene expression in α-factor synchronized cells. In both WT and *set1Δ* mutant, the transcription of histone genes *HTA1* and *HHT2* reached maximal levels at 30 min after α-factor release when S phase occurs (5). It is noteworthy that histone gene expression was significantly reduced in *set1Δ* mutant during S phase (Figure 5B and C), indicating that Set1-catalyzed H3K4me3 regulates histone gene expression primarily at S phase when histone genes are actively transcribed.

The transcription of histone genes *HHT2, HHF2, HTA2*, and *HTB2* was significantly reduced in H3K4 mutants (H3K4A, H3K4Q, H3K4R) (Figure 5D, Supplementary Figure S5D and E). To examine whether Set1-catalyzed H3K4me3 is required for histone gene expression, we examined the transcription of histone genes in *spp1Δ* mutant by qRT-PCR and found the transcription of *HHT2, HHF2, HTA2* and *HTB2* was significantly reduced in *spp1Δ* mutant (Figure 5E). Histone gene transcription was also significantly reduced in H3R2A, H3T6A and H3K14A mutants (Figure 5F–H), consistent with our Western blots results (Figure 3K–N). We also noted that not all histone mutants regulate histone proteins at the transcription level. For example, although H3R40A, H3R72A and H4R55A mutations significantly reduced histone gene transcription, histone gene transcription remained unchanged in H4L37A mutant (Supplementary Figure S5F). Histone gene transcription was not significantly affected in H3D77A and H3D81A mutants (Supplementary Figure S5G), which have higher histone protein levels than WT (Supplementary Figure S4). Altogether, these data indicate that Set1 complex-catalyzed H3K4me3 promotes histone gene transcription primarily at S phase.

### Set1-catalyzed H3K4me3 relieves the repressive effects of HIR/Asf1/Rtt106 on histone gene expression

Next, we explored the mechanism for how Set1-catalyzed H3K4me3 promotes histone gene transcription. Histone acetyltransferase Gcn5 is required for histone gene expression (37). The Gcn5-containing histone acetyltransferase SAGA complex contains Sgf29 that harbors tandem Tudor domains, which selectively bind H3K4me2/3 to facilitate the recruitment of SAGA to acetylate histones in gene promoters (38). One plausible mechanism for Set1-catalyzed H3K4me3 to promote histone gene expression is recruiting Gcn5-containing histone acetyltransferase SAGA to acetylate histones at histone gene promoters. However, we did not observe any significant effect of Sgf29 on histone gene expression (Supplementary Figure S6A and B). Set1-catalyzed H3K4 methylation has been reported to regulate gene expression via histone deacetylation (39,40). Set1 has also been reported to regulate gene expression by interacting with histone deacetylases (HDACs) (41). To investigate whether Set1-catalyzed H3K4me3 regulates histone gene transcription via HDACs, we analyzed histone protein levels in deletion mutants of all known HDACs in yeast including Hos1, Hos2, Hos3, Hos4, Sir2, Rpd3, Hst2, Hst3, Hst4 and Hda1. Our data showed that these HDACs have no dramatic effect on histone gene expression (Supplementary Figure S6C and D). Treatment of WT and H3K4A mutant with HDACs inhibitors (trichostain A (TSA), nicotinamide (NAM), sodium butyrate) also failed to restore the reduced histone proteins in H3K4A mutant (Supplementary Figure S6E–G). Moreover, Set1-catalyzed H3K4me3 did not affect H3K56ac (Supplementary Figure S6H). All these data suggest that Set1-catalyzed H3K4me3 promotes histone gene transcription not by affecting histone acetylation levels.

**Figure 6.**
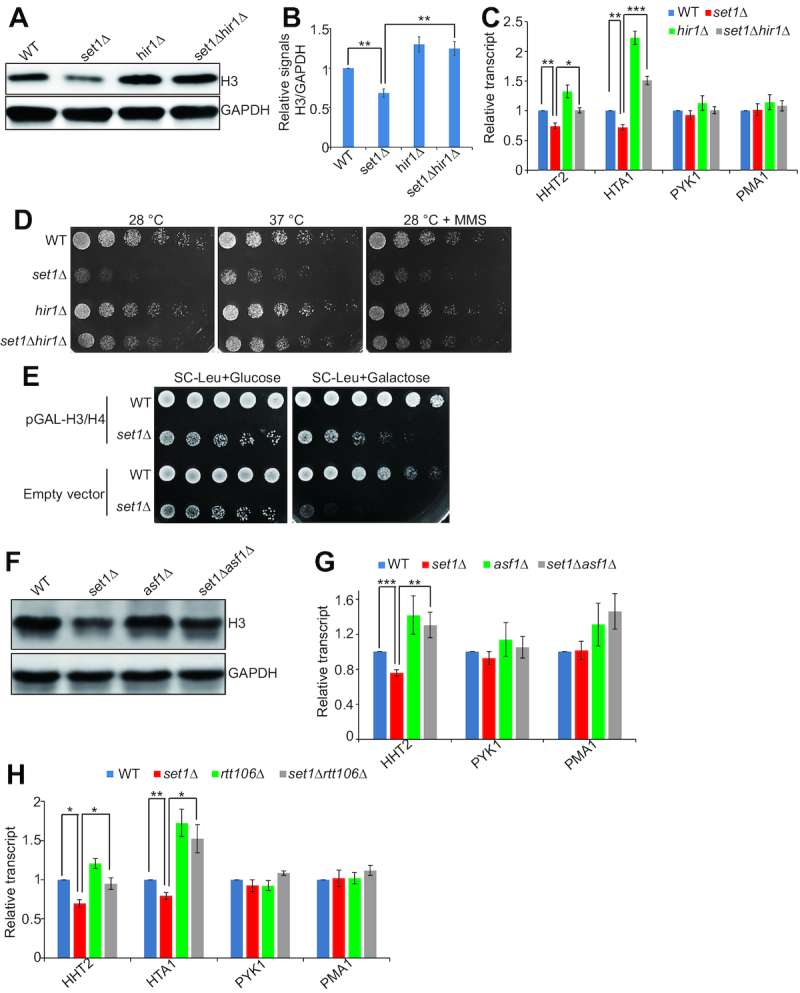
Set1-catalyzed H3K4me3 antagonizes the repressive function of HIR/Asf1/Rtt106 on histone gene expression. (**A** and **B**) Western blots analysis of histone proteins in exponential growing cells (WT, *set1Δ, hir1Δ, set1Δhir1Δ*). (**C**) qRT-PCR analysis of *HTA1, HHT2, PYK1* and *PMA1* transcripts in exponential growing cells (WT, *set1Δ, hir1Δ, set1Δhir1Δ*). The RNA levels of these genes were normalized to *ACTIN*. The transcripts of *PYK1* and *PMA1* were not significantly affected by *SET1* and *HIR1* deletion and served as negative controls. (**D**) Serial diluted WT, *set1Δ, hir1Δ*, and *set1Δhir1Δ* cells were spotted on YPD at 28°C or 37°C or at YPD + methyl methanesulfonate (MMS) at 28°C. Shown is the typical example of three independent experiments. (**E**) Serial diluted WT (empty vector or pGAL-H3/H4), *set1Δ* (empty vector or pGAL-H3/H4) cells were spotted on synthetic complete medium (SC) - leucine (Leu) + 2% glucose and SC – Leu + 2% galactose at 28°C. When cells were grown on SC – Leu + 2% glucose plate, *set1*Δ transformed with pGAL-H3/H4 grew similar to *set1*Δ transformed with empty vector. However, when cells were grown on SC – Leu + 2% galactose plate, *set1*Δ transformed with pGAL-H3/H4 grew much better than *set1*Δ transformed with empty vector. Shown is the typical example of three independent experiments. (**F**) Western blots analysis of histone proteins in exponential growing cells (WT, *set1Δ, asf1Δ, set1Δasf1Δ*). (**G**) qRT-PCR analysis of the transcription of *HHT2, PYK1* and *PMA1* in exponential growing cells (WT, *set1Δ, asf1Δ, set1Δasf1Δ*). (H) qRT-PCR analysis of the transcription of *HTA1, HHT2, PYK1* and *PMA1* in exponential growing cells (WT, *set1Δ, rtt106Δ, set1Δ rtt106Δ*). Data represent the mean ± SE of three independent experiments. (*) *P* < 0.05; (**) *P* < 0.01; (***) *P* < 0.001, unpaired *t*-test two-tailed *P-*value compared with the corresponding wild type.

Histone gene transcription is repressed by HIR H3/H4 histone-protein chaperone complex (Hir1, Hir2, Hir3 and Hpc2) outside of S phase (23,24). We thus investigated whether Set1-catalyzed H3K4me3 could alleviate the repressive effect of HIR complex. Histone H3 protein levels were analyzed in WT, *set1Δ, hir1Δ* and *set1Δhir1Δ* mutants by Western blots. The significant reduced histone proteins in *set1Δ* mutant was restored in *set1Δhir1Δ* mutant (Figure 6A and B). qRT-PCR analysis showed that the transcription of histone genes, *HHT2* and *HTA1* was significantly reduced in *set1Δ* mutant but it was rescued in *set1Δhir1Δ* mutant as their transcription was significantly higher in *set1Δhir1Δ* mutant than that in *set1Δ* mutant (Figure 6C). *HTA2-HTB2* has no NEG region and deletion of *HIR1* failed to rescue the reduced expression of *HTA2* and *HTB2* in *set1Δ* mutant (Supplementary Figure S6I), indicating that deletion of *HIR1* compensates for the reduced expression of three histone loci (*HTB1-HTA1, HHT1-HHF1* and *HHT2-HHF2*) in *set1Δ* mutant. Deletion of *HIR1* cannot rescue the reduced histone gene expression in some histone mutants, such as H3R40A (Supplementary Figure S6J). Consistent with the above data, deletion of *HIR1* partly restored the slow growth of *set1Δ* mutant (Figure 6D), indicating a genetic interaction between Set1 and the HIR complex. As Hir1 has many other functions in addition to repressing histone gene expression (42), we also investigated whether ectopic expression of extra histones could rescue the slow growth of *set1Δ* mutant. Ectopic expression of histones in *set1Δ* mutant grew much better than *set1Δ* mutant transformed with empty vector when grown on galactose-containing medium (Figure 6E, Supplementary Figure S6K).

The HIR complex requires the H3–H4 histone chaperones, Asf1 and Rtt106 to repress histone gene expression (14,23,43). We thus examined whether deletion of *ASF1* and *RTT106* could rescue histone gene expression in *set1Δ* mutant. *set1Δasf1Δ* mutant has higher histone levels than *set1Δ* mutant (Figure 6F). The qRT-PCR results showed that the transcription of *HHT2* in *set1Δasf1Δ* mutant was significantly higher than that in *set1Δ* mutant (Figure 6G). Similar to *set1Δasf1Δ* mutant, *set1Δrtt106Δ* mutant has significantly higher histone gene transcription than *set1Δ* mutant (Figure 6H). These data indicate that Set1-catalyzed H3K4me3 promotes the transcription of *HTB1-HTA1, HHT1-HHF1* and *HHT2-HHF2* by antagonizing the repressive function of HIR/Asf1/Rtt106.

### Set1-catalyzed H3K4me3 acts as the boundary to restrict the spread of Rtt106 to histone coding regions

**Figure 7.**
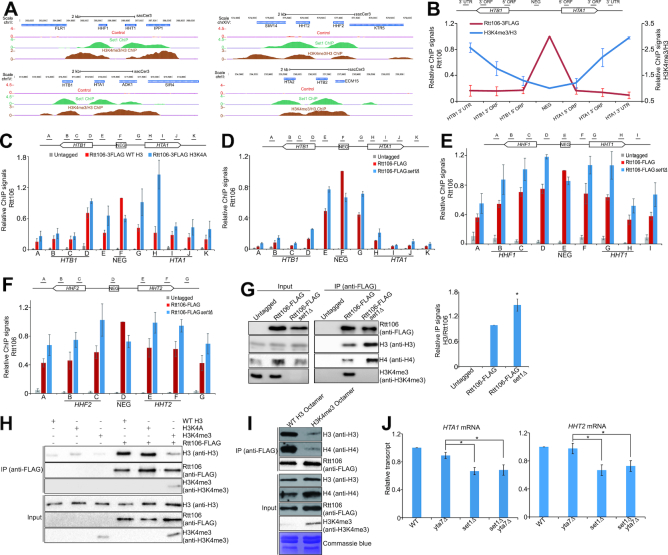
Set1-catalyzed H3K4me3 functions as a boundary to reduce the spread of Rtt106 to histone gene coding regions. (**A**) ChIP-seq analysis of the localization of Set1 and H3K4me3/H3 at *HHF1-HHT1, HHT2-HHF2, HTB1-HTA1*, and *HTA2-HTB2* in exponential growing WT cells. The ChIP-seq for H3K4me3/H3 has been done three biological replicates and only one replicate (log_2_(H3K4me3/H3)) was shown here. The Set1 ChIP-seq data (log_2_(IP/Input)) were retrieved from GSE72972. All replicates for H3K4me3/H3 and Set1 ChIP-seq data were shown in Supplementary Figure 7. (**B**) ChIP-qPCR analysis of the binding of Rtt106 and H3K4me3/H3 at *HTB1-HTA1* in exponential growing WT cells. The data of Rtt106 were presented as the ratio of Rtt106 ChIP signal over input signal. The data of H3K4me3/H3 were presented as the ratio of H3K4me3 ChIP signal over H3 ChIP signal. The error bars represent SE of three independent replicates. (**C**) ChIP-qPCR analysis of the binding of Rtt106 at *HTB1-HTA1* in exponential growing cells (WT H3, H3K4A). (D–F) ChIP-qPCR analysis of the binding of Rtt106 at *HTB1-HTA1* (**D**), *HHT1-HHF1* (**E**) and *HHT2-HHF2* (**F**) in exponential growing cells (WT, *set1Δ*). (**G**) *In vivo* Co-IP assay showing that Set1 inhibits the interaction between Rtt106 and histones. Rtt106 was immunoprecipitated from cell lysates of untagged, Rtt106-FLAG, and Rtt106-FLAG *set1Δ* strains with anti-FLAG antibody. The co-IPed histones were detected by anti-H3, anti-H4 and anti-H3K4me3 antibodies. The relative IP signals of H3/Rtt106 were quantitated in the right panel. (**H**) *In vitro* Co-IP assay showing that H3K4me3 inhibits the interaction between Rtt106 and histone H3. Purified FLAG tagged Rtt106 (Rtt106-FLAG) was incubated with purified recombinant histones H3, H3K4A and H3K4me3. Rtt106-FLAG was immunoprecipitated with anti-FLAG antibody and co-IPed histones were detected by anti-H3 and anti-H3K4me3 antibodies. As a mock IP control, purified recombinant histones H3, H3K4A and H3K4me3 were incubated with anti-FLAG beads without Rtt106-FLAG. (**I**) *In vitro* Co-IP assay with recombinant octamers showing that H3K4me3 inhibits the interaction between Rtt106 and octamers. Purified FLAG tagged Rtt106 (Rtt106-FLAG) was incubated with purified recombinant WT octamer and H3K4me3 octamer. Rtt106-FLAG was immunoprecipitated with anti-FLAG antibody and co-IPed octamers were detected by anti-H3, anti-H4 and anti-H3K4me3 antibodies. (**J**) qRT-PCR analysis of the transcription of *HTA1* and *HHT2* in exponential growing cells (WT, *set1Δ, yta7Δ, set1Δyta7Δ*). The RNA levels of these two genes were normalized to *ACTIN*. Data represent the mean ± SE of three independent experiments. (*) *P* < 0.05.

To show that Set1-catalyzed H3K4me3 directly regulates histone gene transcription, we performed chromatin immunoprecipitation (ChIP)-seq analysis for H3K4me3. Our data showed that H3K4me3 were enriched in all histone gene loci *HHT1-HHF1, HHT2-HHF2, HTB1-HTA1* and *HTA2-HTB2* (Figure 7A, Supplementary Figure S7). Set1 was also enriched in all histone gene loci (Figure 7A, Supplementary Figure S7). In contrast to the binding pattern of Rtt106, which preferentially bound to NEG regions (Figure 7B), Set1 and H3K4me3 preferentially located at histone gene coding regions but not at NEG regions (Figure 7A and B, Supplementary Figure S8A), suggesting that Set1-catalyzed H3K4me3 and Rtt106 could have opposite functions in regulating histone gene expression. Set1-catalyzed H3K4me3 has no remarkable effect on Rtt106 global protein levels (Supplementary Figure S8B–D). We thus examined the effect of Set1-catalyzed H3K4me3 on localization of Rtt106 at histone gene loci. In H3K4A mutant, the enrichment of Rtt106 was reduced at *HTB1-HTA1* NEG region but remarkably increased at regions close to and at *HTB1* and *HTA1* open reading frames (ORFs) (Figure 7C). We also observed increased Rtt106 enrichment in *HTB1-HTA1, HHT1-HHF1* and *HHT2-HHF2* gene loci in *set1Δ* mutant (Figure 7D–F). Set1-catalyzed H3K4me3 did not affect the binding of HIR complex at histone gene loci (Supplementary Figure S8E and F), indicating that Set1-catalyzed H3K4me3 primarily restricts the spreading of Rtt106 from the NEG regions of three HIR-dependent histone gene loci.

To further show that Set1-catatlyzed H3K4me3 functions as a boundary to restrict the spread of Rtt106, we examined the effect of H3K4me3 on interaction between Rtt106 and histones by co-immunoprecipitation (Co-IP) assay. Rtt106 was individually immunoprecipitated from cell extracts of Rtt106-FLAG and Rtt106-FLAG *set1Δ* mutant by anti-FLAG resin and co-IPed histones were detected by anti-H3 and anti-H4 antibodies. As shown in Figure 7G, deletion of *SET1* significantly enhanced the binding of Rtt106 to histone H3 and H4 (Figure 7G), indicating Set1 inhibits the interaction between Rtt106 and histones. We also performed *in vitro* Co-IP assay by incubating purified FLAG tagged Rtt106 with purified recombinant histones (WT H3, H3K4A and H3K4me3) and Rtt106 was then immunoprecipitated by anti-FLAG resin. The Co-IP assay showed that H3K4me3 directly inhibited the interaction between Rtt106 and histones (Figure 7H). To further examine whether H3K4me3 modulates the interaction between Rtt106 and chromatin, we performed *in vitro* Co-IP assay with purified recombinant octamers. The results showed that Rtt106 pulled down more WT octamer than H3K4me3 octamer (Figure 7I). Combined with our ChIP data (Figure 7C–F), the *in vivo* and *in vitro* Co-IP results indicate that Set1-catalyzed H3K4me3 directly restricts the spread of Rtt106 from histone gene promoters to coding regions by disrupting the binding of Rtt106 to histones.

To investigate whether Set1-catalzyed H3K4me3 regulates histone gene expression by altering chromatin structure at histone genes, we examined histone H3 occupancy in *HHT1-HHF1* and *HTA1-HTB1* loci in both WT and *set1Δ* mutant. However, histone H3 occupancy at both *HHT1-HHF1* and *HTA1-HTB1* loci was unaffected by *SET1* deletion (Supplementary Figure S8G), excluding the possibility that Set1 indirectly regulates histone gene expression by regulating chromatin structures.

Yta7 has been reported to function as a boundary protein to restrict the spread of Rtt106 into gene coding regions to repress histone gene expression outside of S phase (14,17). In *yta7Δ* mutant, the enrichment of Rtt106 has been reported to be increased at the promoters and 5′ ORFs of histone genes (17). Since Set1 and Yta7 have similar boundary function in restricting Rtt106 localization, we thus examined whether Set1 and Yta7 act in the same pathway or distinct pathways. The transcription of *HTA1* and *HHT2* in *set1Δyta7Δ* mutant was similar to that in *set1Δ* but was much lower than that in *yta7Δ* mutant (Figure 7J), indicating that Set1 functions as the primary boundary proteins in regulating histone gene expression. Overall, these data indicate that Set1-catalyzed H3K4me3 promotes histone gene expression by restricting the spread of Rtt106 from histone gene NEG regions into their respective coding regions.

## DISCUSSION

Aging is the major risk factor for many human malignancies. However, it is largely unknown about the mechanisms that regulate aging at the cellular level. The aging-associated histone loss was observed in mouse muscle stem cells during chronological aging, but there was no evidence to support their causal relevance due to technical challenges (44). By taking advantage of yeast genetics, we showed in this study that reduced histone protein is a cause of chronological aging and increasing histone supply can extend chronological life span. Using histone mutants, H3D77A and H3D81A, we showed that it is important to maintain proper but not excessive histone protein levels for healthy life span.

Although maintaining proper histone protein levels is important for longevity, little is known about how histone genes are regulated, especially from the perspective of histone posttranslational modifications. Histone acetylation, i.e. H3K56ac has been reported to positively regulate histone gene expression (25). Histone H2BY37 phosphorylation inhibits histone H2B transcription (26). In this study, we screened the histone H3/H4 mutant library for histone gene regulators and identified a total of 20 substitution mutations that have altered histone proteins. Among these 20 substitution mutants, 15 substitutions have reduced histone proteins and most mutations occurred on lysine or arginine residues (Figure 2C). It is likely that the positive charges of these residues or modifications of these residues are required for histone gene expression. Among these residues, H3R2 and H3K4 can be methylated; H3K14 and H3K56 can be acetylated in budding yeast. Our study for the first time showed that Set1-catalyzed H3K4me3 promotes histone gene expression. The effects of H3R2A, H3T6A and H3K14A on histone gene expression could be primarily due to their impact on H3K4me3. The corresponding modifications could be H3R2 asymmetric dimethylation (H3R2me2a), H3T6 phosphorylation (H3T6ph) and H3K14 acetylation (H3K14ac) as these modifications have been reported to regulate H3K4me3 (29,30,35). H3R17 has been shown to undergo asymmetric dimethylation in mammals but it is unknown whether it can be modified in yeast (45). H3R49 and H3R53 are close to H3K56 and they regulate histone gene expression probably by affecting H3K56ac (Figure 2D). H4K44 could be acetylated to increase the dynamic accessibility of the nucleosomal DNA, similar to H3K56ac (46). However, it should be noted that our screening is based on histone protein levels, thus it is possible that these mutants may have pleiotropic effects and may not regulate histone gene transcription directly, such as H4L37A, H3D77A and H3D81A. Further efforts are required to figure out how these mutants regulate histone proteins.

Our data showed that H3K4me3 promotes histone expression and maintains normal chronological life span. While loss of H3K4me3 leads to shortened chronological life span (Figure 4A and B), it has been reported that H3K4R, D, E, Q mutants have no effect on replicative life span (47), implying that chronological life span and replicative life span are not always controlled by the same pathway.

**Figure 8.**
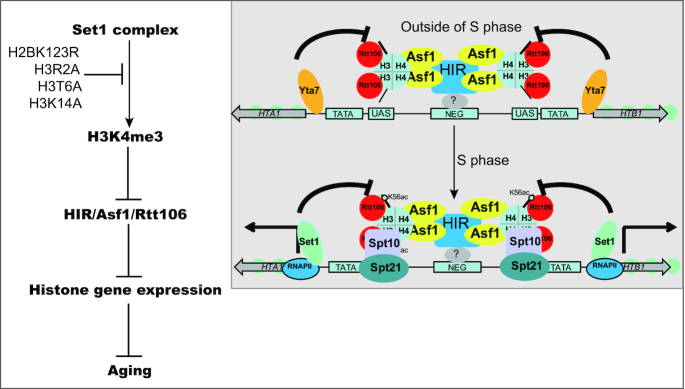
Diagram showing Set1-catalyzed H3K4me3 maintains normal chronological life span by promoting histone gene expression. Inserted small box is the diagram showing Set1-catalyzed H3K4me3 promotes histone gene expression by functioning as the boundary at S phase. When cells are outside of S phase, Yta7 functions as the boundary to reduce the spreading of HIR/Asf1/Rtt106 complex to histone coding regions. When cells enter S phase, transcription activators, i.e. Spt10 and Spt21 bind to the UAS regions. Yta7 is phosphorylated and dissociated from histone gene loci and Set1 functions as the primary boundary to prevent the spreading the HIR/Asf1/Rtt106 complex. By promoting histone gene expression, Set1-catalyzed H3K4me3 is important to maintain normal chronological life span. In mutants that have compromised H3K4me3, histone gene expression is reduced and life span is shortened.

Our study reveals a novel mechanism by which Set1-catalyzed H3K4me3 regulates histone gene expression. Fillingham *et al.* have reported that histone gene activation requires relief of Rtt106-mediated repression by Rtt109 and restriction of the spread of Rtt106 to gene coding regions by Yta7 (14). Set1-catalyzed H3K4me3 has no effect on Rtt109-catalyzed H3K56ac (Supplementary Figure S6H), excluding the involvement of Rtt109. Meanwhile, Set1-catalyzed H3K4me3 has no effect on the chromatin structure at histone genes (Supplementary Figure S8G). Although we cannot rule out all possible pleiotropic effects of H3K4me3, the major role of Set1-catalyzed H3K4me3 in histone gene expression is preventing the spreading of Rtt106. Moreover, H3K4me3 could function as a major boundary element in regulating histone gene expression based on our qRT-PCR analysis of *set1Δyta7Δ* (Figure 7J). Kurat *et al.* reported that Yta7 is phosphorylated by cyclin-dependent kinase 1 and casein kinase 2 during S phase, which leads to its release from histone *HTA1* coding regions to facilitate RNA polymerase II elongation (18). Their data clearly indicate that Yta7 functions as a boundary protein only at G1, G1/S and M phases when histone genes are repressed. During S phase when histone genes are actively transcribed, Yta7 impairs efficient transcription and needs to be phosphorylated and dissociated from chromatin, which makes it impossible to function as a boundary protein during S phase. As Rtt106-containing repressive complex HIR/Asf1/Rtt106 binds at histone loci throughout all stages of cell cycles (43), Set1-catalyzed H3K4me3 could function as the boundary to prevent the spread of the repressive complex during S phase. Indeed, our qRT-PCR data showed that Set1-catalyzed H3K4me3 regulates histone gene expression primarily during S phase when histone genes are transcribed (Figure 5B and C). Hence, the reported data and our observations revealed that there are two boundary elements in histone gene expression: Set1-catalyzed H3K4me3 functions at S phase and Yta7 functions outside of S phase (Figure 8). Histone H3K4me3 is highly conserved in higher organisms. It will be interesting to investigate whether H3K4me3 and its regulatory histone PTMs (H3R2me2a, H3T6ph and H3K14ac) regulate histone gene expression in these organisms by the same mechanism.

Our data showed that Set1-catalyzed H3K4me3 also promotes the transcription of *HTA2* and *HTB2*, which contain no NEG region. Moreover, deletion of *HIR1* cannot rescue the reduced *HTA2* and *HTB2* expression in *set1Δ* mutant. Our results thus indicate that H3K4me3 promotes the transcription of *HTA2* and *HTB2* in a mechanism independent of HIR/Asf1/Rtt106 repressive complex. H2B Tyr40 phosphorylation has been shown to repress histone gene expression by facilitating the binding of HIRA, the homolog of HIR complex in mammals (26). Interestingly, H2B Tyr40 phosphorylation also represses the transcription of *HTA2* and *HTB2* (26). It is possible that Set1-catalyzed H3K4me3 promotes *HTA2* and *HTB2* expression by regulating the binding of other histone regulators, such as Spt10 and Spt21. Deletion of *SPT10* or *SPT21* has a more dramatic effect on the transcription of *HTA2* and *HTB2* compared with other histone genes (48). Moreover, Spt10 and Spt21 bind to the *HTA2-HTB2* promoter during S phase to activate histone gene expression. At the end of S phase, Spt10 and Spt21 are displaced from the *HTA2-HTB2* promoter to stop gene transcription. As H3K4me3 promotes histone gene expression primarily at S phase, it is possible that H3K4me3 may regulate the binding of Spt10 and Spt21. Further efforts are required to address this question.

How does Set1-catalyzed H3K4me3 inhibit the spread of Rtt106? One possibility is that H3K4me3-specific ‘readers’ are recruited to prevent the spread of Rtt106. We initially thought the factor could be Tudor domain-containing Sgf29, which has been shown to preferentially bind H3K4me3 (38). However, histone gene expression was not reduced in the *sgf29Δ* mutant (Supplementary Figure S6A and B), which excludes this possibility. Our *in vitro* Co-IP data indicate that purified recombinant histone H3K4me3 interferes the binding of Rtt106 to histones even in the absence of H3K56ac (Figure 7H). Thus, these data demonstrate that H3K4me3 itself but not its ‘readers’ serves as the boundary to restrict the spread of Rtt106 from NEG regions to neighbouring coding regions. Rtt106 contains two nucleosome binding sites: one located in its N-terminal dimerization domain, which binds H3-H4 tetramers in an H3K56ac-independent manner; the other one located in its double PH domain, which binds H3-H4 tetramers in a H3K56ac-dependent manner (49). Given the fact that H3K4me3 inhibits Rtt106 binding to recombinant histone H3 that has no H3K56ac (Figure 7H), it is likely that H3K4me3 interferes the interaction between Rtt106 N-terminal domain and histones.

H3K4me3 has been considered as a universal hallmark of active transcription and there is a strong correlation between active transcription and the occurrence of H3K4me3 around transcriptional start sites (50). However, there is no conserved mechanism to support this view and the functions of H3K4me3 in gene transcription are far from completely understood (50). Transcriptome analysis showed that in *set1Δ* mutant, 69 genes were significantly upregulated and only 20 genes were down-regulated, indicating that Set1 has a more repressive effect on gene expression (51). Similar expression profiles have been observed in Set1 complex mutants, i.e. 57 and 23 genes are significantly upregulated and down-regulated in *spp1Δ* mutant, respectively (51), indicating that Set1 complex-catalyzed H3K4me3 plays a more repressive role in gene transcription. Zhang *et al.* observed the global occurrence of non-canonical broad H3K4me3 coincides with genome silencing from mature oocytes to the early 2-cell stage, suggesting that H3K4me3 may contribute to gene repression in higher organisms (52). Regarding the repressive mechanism, Set1-catalyzed H3K4me3 has been reported to inhibit gene transcription by promoting 3′ end antisense transcription (51). The genes repressed via antisense transcripts are enriched with H3K4me3 at their 3′ coding regions (51). Our data showed that Set1 and H3K4me3 are highly enriched at 3′ coding regions of histone genes (Figure 7A and B, Supplementary Figure S8A), which are activated by Set1-catalyzed H3K4me3. Moreover, stabilization of the antisense transcripts by deletion of the exosome component *RRP6* does not have a general effect on Set1-repressed genes (51). Thus, antisense transcripts may not explain the repressive effect of Set1 on genome-wide gene transcription. Our data suggest that the upregulated gene expression in *set1Δ* mutant could be caused by reduced histone expression and probably more open chromatin structure, thereby providing a novel and general mechanism for how Set1-catalyzed H3K4me3 mediates genome-wide gene repression.

Collectively, we presented here a systematic analysis of histone gene regulation and chronological life span regulation from the perspectives of histone posttranslational modifications. We uncovered a novel mechanism by which H3K4me3 promotes histone gene transcription, providing a plausible explanation for genome-wide repression by H3K4me3. Moreover, we uncovered the underlying mechanism linking histone gene expression and chronological life span by Set1-catalyzed H3K4me3 and its regulatory histone PTMs.

## DATA AVAILABILITY

The GEO accession number for the raw ChIP-seq dataset in this paper is GEO: GSE115910. The ChIP-seq data for Set1 were retrieved from GSE81822 and GSE72972.

## Supplementary Material

Supplementary Data

## References

[1] Lopez-Otin C., Blasco M.A., Partridge L., Serrano M., Kroemer G. The hallmarks of aging. Cell. 2013; 153:1194–1217. 23746838 10.1016/j.cell.2013.05.039 PMC3836174

[2] Longo V.D., Shadel G.S., Kaeberlein M., Kennedy B. Replicative and chronological aging in *Saccharomyces cerevisiae*. Cell Metab. 2012; 16:18–31. 22768836 10.1016/j.cmet.2012.06.002 PMC3392685

[3] Campos S.E., Avelar-Rivas J.A., Garay E., Juarez-Reyes A., DeLuna A. Genomewide mechanisms of chronological longevity by dietary restriction in budding yeast. Aging Cell. 2018; 17:e12749. 29575540 10.1111/acel.12749 PMC5946063

[4] Luger K., Mader A.W., Richmond R.K., Sargent D.F., Richmond T.J. Crystal structure of the nucleosome core particle at 2.8 A resolution. Nature. 1997; 389:251–260. 9305837 10.1038/38444

[5] Kurat C.F., Lambert J.P., Petschnigg J., Friesen H., Pawson T., Rosebrock A., Gingras A.C., Fillingham J., Andrews B. Cell cycle-regulated oscillator coordinates core histone gene transcription through histone acetylation. Proc. Natl. Acad. Sci. U.S.A. 2014; 111:14124–14129. 25228766 10.1073/pnas.1414024111 PMC4191790

[6] Nelson D.M., Ye X., Hall C., Santos H., Ma T., Kao G.D., Yen T.J., Harper J.W., Adams P.D. Coupling of DNA synthesis and histone synthesis in S phase independent of cyclin/cdk2 activity. Mol. Cell. Biol. 2002; 22:7459–7472. 12370293 10.1128/MCB.22.21.7459-7472.2002 PMC135676

[7] Han M., Chang M., Kim U.J., Grunstein M. Histone H2B repression causes cell-cycle-specific arrest in yeast: effects on chromosomal segregation, replication, and transcription. Cell. 1987; 48:589–597. 3815518 10.1016/0092-8674(87)90237-6

[8] Prado F., Aguilera A. Partial depletion of histone H4 increases homologous recombination-mediated genetic instability. Mol. Cell. Biol. 2005; 25:1526–1536. 15684401 10.1128/MCB.25.4.1526-1536.2005 PMC548009

[9] Celona B., Weiner A., Di Felice F., Mancuso F.M., Cesarini E., Rossi R.L., Gregory L., Baban D., Rossetti G., Grianti P. et al. Substantial histone reduction modulates genomewide nucleosomal occupancy and global transcriptional output. PLoS Biol. 2011; 9:e1001086. 21738444 10.1371/journal.pbio.1001086 PMC3125158

[10] Kurat C.F., Recht J., Radovani E., Durbic T., Andrews B., Fillingham J. Regulation of histone gene transcription in yeast. Cell. Mol. Life Sci. 2014; 71:599–613. 23974242 10.1007/s00018-013-1443-9 PMC11113579

[11] Mei Q., Huang J., Chen W., Tang J., Xu C., Yu Q., Cheng Y., Ma L., Yu X., Li S. Regulation of DNA replication-coupled histone gene expression. Oncotarget. 2017; 8:95005–95022. 29212286 10.18632/oncotarget.21887 PMC5706932

[12] Iyer V.R., Horak C.E., Scafe C.S., Botstein D., Snyder M., Brown P.O. Genomic binding sites of the yeast cell-cycle transcription factors SBF and MBF. Nature. 2001; 409:533–538. 11206552 10.1038/35054095

[13] Eriksson P.R., Ganguli D., Clark D.J. Spt10 and Swi4 control the timing of histone H2A/H2B gene activation in budding yeast. Mol. Cell. Biol. 2011; 31:557–572. 21115727 10.1128/MCB.00909-10 PMC3028627

[14] Fillingham J., Kainth P., Lambert J.P., van Bakel H., Tsui K., Pena-Castillo L., Nislow C., Figeys D., Hughes T.R., Greenblatt J. et al. Two-color cell array screen reveals interdependent roles for histone chaperones and a chromatin boundary regulator in histone gene repression. Mol. Cell. 2009; 35:340–351. 19683497 10.1016/j.molcel.2009.06.023

[15] Ng H.H., Robert F., Young R.A., Struhl K. Genome-wide location and regulated recruitment of the RSC nucleosome-remodeling complex. Genes Dev. 2002; 16:806–819. 11937489 10.1101/gad.978902 PMC186327

[16] Prochasson P., Florens L., Swanson S.K., Washburn M.P., Workman J.L. The HIR corepressor complex binds to nucleosomes generating a distinct protein/DNA complex resistant to remodeling by SWI/SNF. Genes Dev. 2005; 19:2534–2539. 16264190 10.1101/gad.1341105 PMC1276727

[17] Zunder R.M., Rine J. Direct interplay among histones, histone chaperones, and a chromatin boundary protein in the control of histone gene expression. Mol. Cell. Biol. 2012; 32:4337–4349. 22907759 10.1128/MCB.00871-12 PMC3486138

[18] Kurat C.F., Lambert J.P., van Dyk D., Tsui K., van Bakel H., Kaluarachchi S., Friesen H., Kainth P., Nislow C., Figeys D. et al. Restriction of histone gene transcription to S phase by phosphorylation of a chromatin boundary protein. Genes Dev. 2011; 25:2489–2501. 22156209 10.1101/gad.173427.111 PMC3243059

[19] Hu Z., Chen K., Xia Z., Chavez M., Pal S., Seol J.H., Chen C.C., Li W., Tyler J.K. Nucleosome loss leads to global transcriptional up-regulation and genomic instability during yeast aging. Genes Dev. 2014; 28:396–408. 24532716 10.1101/gad.233221.113 PMC3937517

[20] Feser J., Truong D., Das C., Carson J.J., Kieft J., Harkness T., Tyler J.K. Elevated histone expression promotes life span extension. Mol. Cell. 2010; 39:724–735. 20832724 10.1016/j.molcel.2010.08.015 PMC3966075

[21] Li S., Swanson S.K., Gogol M., Florens L., Washburn M.P., Workman J.L., Suganuma T. Serine and SAM responsive complex SESAME regulates histone modification crosstalk by sensing cellular metabolism. Mol. Cell. 2015; 60:408–421. 26527276 10.1016/j.molcel.2015.09.024

[22] Yu Q., Tong C., Luo M., Xue X., Mei Q., Ma L., Yu X., Mao W., Kong L., Yu X. et al. Regulation of SESAME-mediated H3T11 phosphorylation by glycolytic enzymes and metabolites. PLoS One. 2017; 12:e0175576. 28426732 10.1371/journal.pone.0175576 PMC5398556

[23] Green E.M., Antczak A.J., Bailey A.O., Franco A.A., Wu K.J., Yates J.R. 3rd, Kaufman P.D. Replication-independent histone deposition by the HIR complex and Asf1. Curr. Biol. 2005; 15:2044–2049. 16303565 10.1016/j.cub.2005.10.053 PMC2819815

[24] Osley M.A., Lycan D. Trans-acting regulatory mutations that alter transcription of *Saccharomyces cerevisiae* histone genes. Mol. Cell. Biol. 1987; 7:4204–4210. 3125420 10.1128/mcb.7.12.4204-4210.1987 PMC368101

[25] Xu F., Zhang K., Grunstein M. Acetylation in histone H3 globular domain regulates gene expression in yeast. Cell. 2005; 121:375–385. 15882620 10.1016/j.cell.2005.03.011

[26] Mahajan K., Fang B., Koomen J.M., Mahajan N.P. H2B Tyr37 phosphorylation suppresses expression of replication-dependent core histone genes. Nat. Struct. Mol. Biol. 2012; 19:930–937. 22885324 10.1038/nsmb.2356 PMC4533924

[27] Dai J., Hyland E.M., Yuan D.S., Huang H., Bader J.S., Boeke J.D. Probing nucleosome function: a highly versatile library of synthetic histone H3 and H4 mutants. Cell. 2008; 134:1066–1078. 18805098 10.1016/j.cell.2008.07.019 PMC2701395

[28] Smolle M., Workman J.L. Transcription-associated histone modifications and cryptic transcription. Biochim. Biophys. Acta. 2013; 1829:84–97. 22982198 10.1016/j.bbagrm.2012.08.008 PMC5854953

[29] Kirmizis A., Santos-Rosa H., Penkett C.J., Singer M.A., Vermeulen M., Mann M., Bahler J., Green R.D., Kouzarides T. Arginine methylation at histone H3R2 controls deposition of H3K4 trimethylation. Nature. 2007; 449:928–932. 17898715 10.1038/nature06160 PMC3350864

[30] Maltby V.E., Martin B.J., Brind’Amour J., Chruscicki A.T., McBurney K.L., Schulze J.M., Johnson I.J., Hills M., Hentrich T., Kobor M.S. et al. Histone H3K4 demethylation is negatively regulated by histone H3 acetylation in *Saccharomyces cerevisiae*. Proc. Natl. Acad. Sci. U.S.A. 2012; 109:18505–18510. 23091032 10.1073/pnas.1202070109 PMC3494965

[31] Shilatifard A. The COMPASS family of histone H3K4 methylases: mechanisms of regulation in development and disease pathogenesis. Annu. Rev. Biochem. 2012; 81:65–95. 22663077 10.1146/annurev-biochem-051710-134100 PMC4010150

[32] van Leeuwen F., Gafken P.R., Gottschling D.E. Dot1p modulates silencing in yeast by methylation of the nucleosome core. Cell. 2002; 109:745–756. 12086673 10.1016/s0092-8674(02)00759-6

[33] Lee J.S., Shukla A., Schneider J., Swanson S.K., Washburn M.P., Florens L., Bhaumik S.R., Shilatifard A. Histone crosstalk between H2B monoubiquitination and H3 methylation mediated by COMPASS. Cell. 2007; 131:1084–1096. 18083099 10.1016/j.cell.2007.09.046

[34] Schulze J.M., Hentrich T., Nakanishi S., Gupta A., Emberly E., Shilatifard A., Kobor M.S. Splitting the task: Ubp8 and Ubp10 deubiquitinate different cellular pools of H2BK123. Genes Dev. 2011; 25:2242–2247. 22056669 10.1101/gad.177220.111 PMC3219228

[35] Metzger E., Imhof A., Patel D., Kahl P., Hoffmeyer K., Friedrichs N., Muller J.M., Greschik H., Kirfel J., Ji S. et al. Phosphorylation of histone H3T6 by PKCbeta(I) controls demethylation at histone H3K4. Nature. 2010; 464:792–796. 20228790 10.1038/nature08839

[36] Ramakrishnan S., Pokhrel S., Palani S., Pflueger C., Parnell T.J., Cairns B.R., Bhaskara S., Chandrasekharan M.B. Counteracting H3K4 methylation modulators Set1 and Jhd2 co-regulate chromatin dynamics and gene transcription. Nat. Commun. 2016; 7:11949. 27325136 10.1038/ncomms11949 PMC4919544

[37] Petty E.L., Lafon A., Tomlinson S.L., Mendelsohn B.A., Pillus L. Promotion of cell viability and histone gene expression by the acetyltransferase Gcn5 and the protein phosphatase PP2A in *Saccharomyces cerevisiae*. Genetics. 2016; 203:1693–1707. 27317677 10.1534/genetics.116.189506 PMC4981271

[38] Bian C., Xu C., Ruan J., Lee K.K., Burke T.L., Tempel W., Barsyte D., Li J., Wu M., Zhou B.O. et al. Sgf29 binds histone H3K4me2/3 and is required for SAGA complex recruitment and histone H3 acetylation. EMBO J. 2011; 30:2829–2842. 21685874 10.1038/emboj.2011.193 PMC3160252

[39] Buratowski S., Kim T. The role of cotranscriptional histone methylations. Cold Spring Harb. Smp. Quant. Biol. 2010; 75:95–102. 10.1101/sqb.2010.75.036 PMC3229092 21447819

[40] Wang S.S., Zhou B.O., Zhou J.Q. Histone H3 lysine 4 hypermethylation prevents aberrant nucleosome remodeling at the *PHO5* promoter. Mol. Cell. Biol. 2011; 31:3171–3181. 21646424 10.1128/MCB.05017-11 PMC3147609

[41] Lorenz D.R., Meyer L.F., Grady P.J., Meyer M.M., Cam H.P. Heterochromatin assembly and transcriptome repression by Set1 in coordination with a class II histone deacetylase. eLife. 2014; 3:e04506. 25497836 10.7554/eLife.04506 PMC4383021

[42] Amin A.D., Vishnoi N., Prochasson P. A global requirement for the HIR complex in the assembly of chromatin. Biochim. Biophys. Acta. 2013; 1819:264–276. 24459729 10.1016/j.bbagrm.2011.07.008

[43] Ferreira M.E., Flaherty K., Prochasson P. The *Saccharomyces cerevisiae* histone chaperone Rtt106 mediates the cell cycle recruitment of SWI/SNF and RSC to the HIR-dependent histone genes. PLoS One. 2011; 6:e21113. 21698254 10.1371/journal.pone.0021113 PMC3115976

[44] Liu L., Cheung T.H., Charville G.W., Hurgo B.M., Leavitt T., Shih J., Brunet A., Rando T.A. Chromatin modifications as determinants of muscle stem cell quiescence and chronological aging. Cell Rep. 2013; 4:189–204. 23810552 10.1016/j.celrep.2013.05.043 PMC4103025

[45] Hatanaka Y., Tsusaka T., Shimizu N., Morita K., Suzuki T., Machida S., Satoh M., Honda A., Hirose M., Kamimura S. et al. Histone H3 methylated at arginine 17 is essential for reprogramming the paternal genome in zygotes. Cell Rep. 2017; 20:2756–2765. 28930672 10.1016/j.celrep.2017.08.088

[46] Fenley A.T., Anandakrishnan R., Kidane Y.H., Onufriev A.V. Modulation of nucleosomal DNA accessibility via charge-altering post-translational modifications in histone core. Epigenetics Chromatin. 2018; 11:11. 29548294 10.1186/s13072-018-0181-5 PMC5856334

[47] Sen P., Dang W., Donahue G., Dai J., Dorsey J., Cao X., Liu W., Cao K., Perry R., Lee J.Y. et al. H3K36 methylation promotes longevity by enhancing transcriptional fidelity. Genes Dev. 2015; 29:1362–1376. 26159996 10.1101/gad.263707.115 PMC4511212

[48] Dollard C., Ricupero-Hovasse S.L., Natsoulis G., Boeke J.D., Winston F. SPT10 and SPT21 are required for transcription of particular histone genes in Saccharomyces cerevisiae. Mol. Cell. Biol. 1994; 14:5223–5228. 8035801 10.1128/mcb.14.8.5223-5228.1994 PMC359041

[49] Su D., Hu Q., Li Q., Thompson J.R., Cui G., Fazly A., Davies B.A., Botuyan M.V., Zhang Z., Mer G. Structural basis for recognition of H3K56-acetylated histone H3-H4 by the chaperone Rtt106. Nature. 2012; 483:104–107. 22307274 10.1038/nature10861 PMC3439842

[50] Howe F.S., Fischl H., Murray S.C., Mellor J. Is H3K4me3 instructive for transcription activation. BioEssays. 2017; 39:1–12. 10.1002/bies.201600095 28004446

[51] Margaritis T., Oreal V., Brabers N., Maestroni L., Vitaliano-Prunier A., Benschop J.J., van Hooff S., van Leenen D., Dargemont C., Geli V. et al. Two distinct repressive mechanisms for histone 3 lysine 4 methylation through promoting 3′-end antisense transcription. PLoS Genet. 2012; 8:e1002952. 23028359 10.1371/journal.pgen.1002952 PMC3447963

[52] Zhang B., Zheng H., Huang B., Li W., Xiang Y., Peng X., Ming J., Wu X., Zhang Y., Xu Q. et al. Allelic reprogramming of the histone modification H3K4me3 in early mammalian development. Nature. 2016; 537:553–557. 27626382 10.1038/nature19361

