# Supplementary material for: Set1-catalyzed H3K4 trimethylation antagonizes the HIR/Asf1/Rtt106 repressor complex to promote histone gene expression and chronological life span
Source: Nucleic Acids Res. 2019 Feb 13;47(7):3434–49. doi: 10.1093/nar/gkz101 (PMC6468302; doi:10.1093/nar/gkz101)
Supplement: Supplementary Data [file gkz101_supplemental_file.pdf]

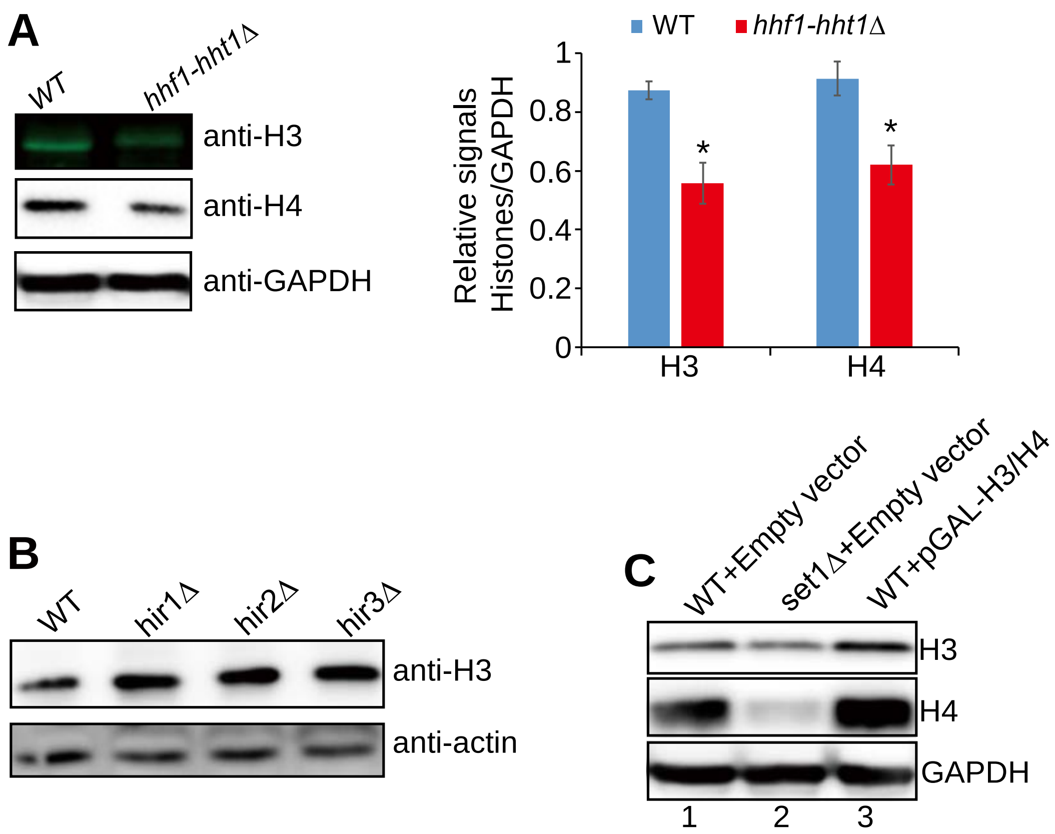

Figure S1. (A) Western blots analysis of histones in WT and *hhf1-hht1Δ* mutant. Yeast cells were grown in YPD until mid-log phase. Cells were harvested, lysed and analyzed by Western blots with indicated antibodies. GAPDH was utilized as an endogenous control. The relative intensities of Histones/ GAPDH were quantified using Image J with standard error (SE). Data represent the mean ± SE of three independent experiments. (*) $P<0.05$. (B) Histones were increased in *hir1Δ, hir2Δ, hir3Δ* mutants by Western blots with indicated antibodies. (C) Strain BY4741 (WT) was transformed with a vector expressing an extra copy of H3 and H4 under the control of the pGAL1/10 divergent promoters (pGAL-H3/H4) (lane 3). WT and *set1Δ* mutant cells transformed with an empty vector were used as controls (lane 1 and lane 2). All cells were grown in SC – leucine + 2% galactose for 3 hrs to induce histone expression. Protein levels were analzyed by Western blots with indicated antibodies. The experiments have been performed triplicates and shown is the typical example.

Supplementary_Fig_S2

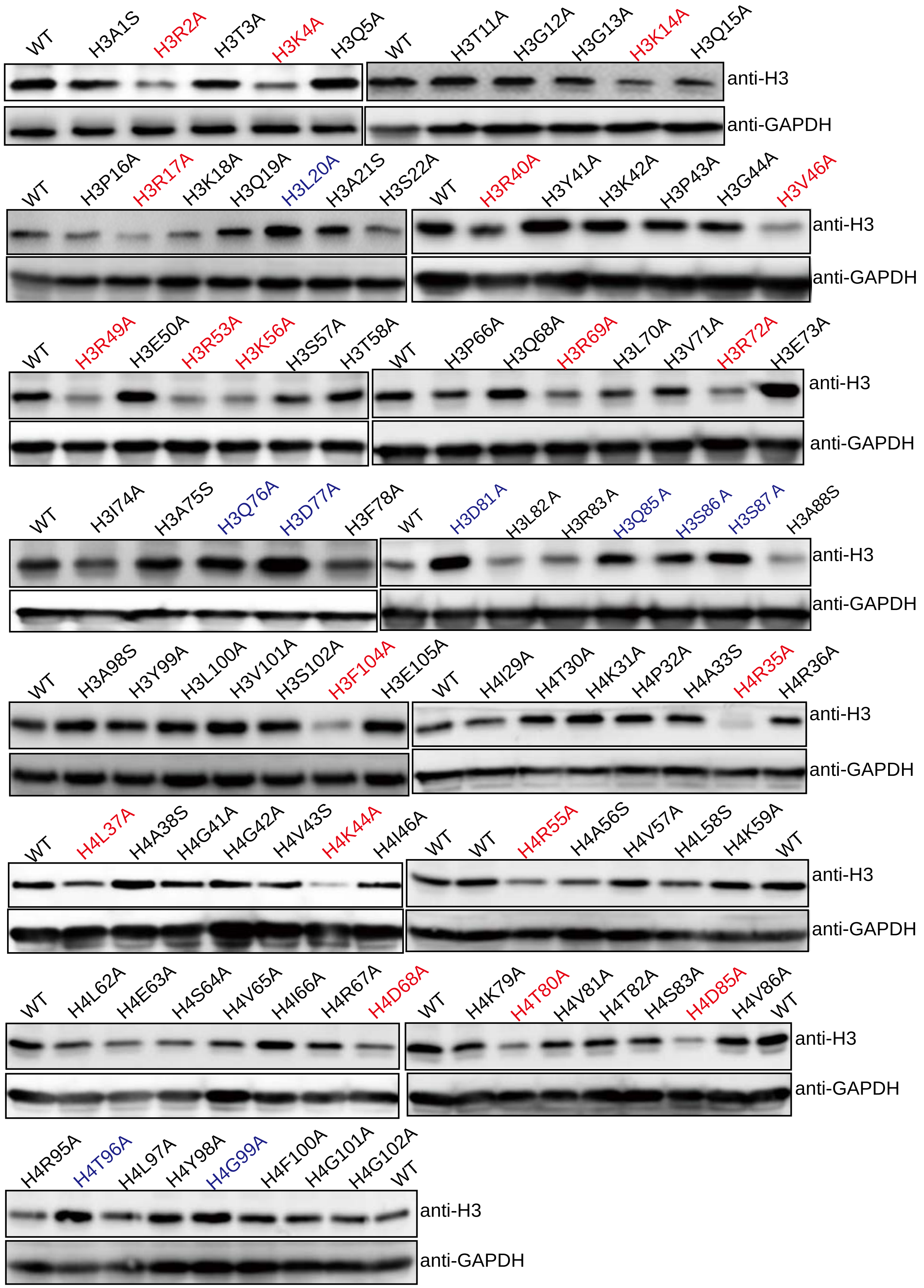

Figure S2. Analysis of H3 protein levels in histone H3/H4 mutant library. Yeast cells (WT and mutants from histone H3/H4 mutant library) were grown in YPD until OD_{600} of 0.7-1.0. Cells were harvested, lysed and analyzed by Western blots with indicated antibodies. GAPDH was utilized as an endogenous control. Only blots containing mutations with 2-fold reduced H3 (in red color) or 1.5-fold increased H3 (in blue color) were shown.

Supplementary_Fig_S3

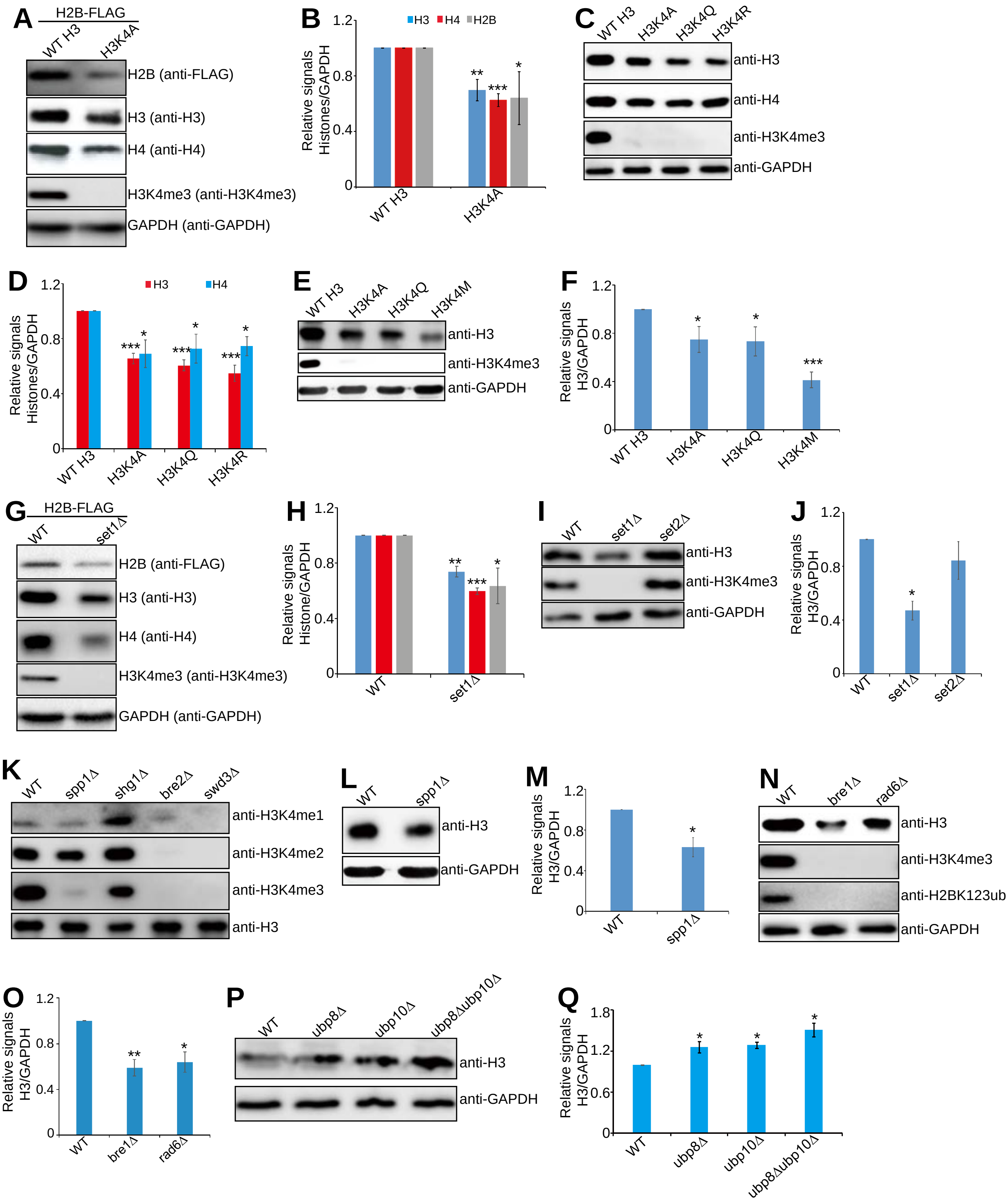

Figure S3. Set1-catalyzed H3K4me3 is required for maintenance of normal intracellular histone proteins. (A and B) Western blots analysis of histone proteins in exponential growing cells (H2B-FLAG WT H3, H2B-FLAG H3K4A) with indicated antibodies. The relative intensities of Histones/GAPDH in Figure S3A were quantified using Image J with standard error (SE) (n=3). (C-F) Western blots analysis of histone proteins in exponential growing cells (WT, H3K4A, H3K4Q, H3K4R, H3K4M) with indicated antibodies. (G and H) Western blots analysis of histone proteins in exponential growing cells (H2B-FLAG WT, H2B-FLAG *set1*Δ) with indicated antibodies. The relative intensities of Histones/GAPDH in Figure S3G were quantified using Image J with standard error (SE) (n=3). (I and J) Western blots analysis of histone proteins in exponential growing cells (WT, *set1*Δ, *set2*Δ). (K) Western blots analysis of H3K4me1, H3K4me2, H3K4me3 in WT, *spp1*Δ, *shg1*Δ, *bre2*Δ, and *swd3*Δ mutants with indicated antibodies. Histone H3 serves as a loading control. (L and M) Western blots analysis of histone H3 in WT and *spp1*Δ mutant with indicated antibodies. (N and O) Western blots analysis of histone proteins in WT, *bre1*Δ, and *rad6*Δ mutants. (P and Q) Western blots analysis of histone H3 in WT, *ubp8*Δ, *ubp10*Δ, and *ubp8*Δ*ubp10*Δ mutants. Data represent the mean ± SE of three independent experiments. (*) $P<0.05$; (***) $P<0.001$.

Supplementary_Fig_S4

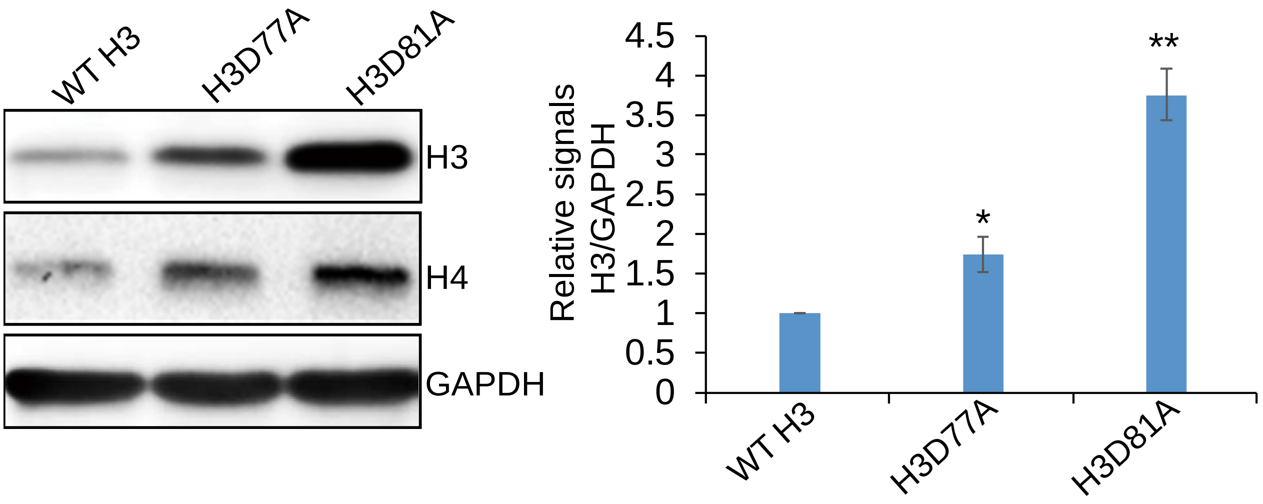

Figure S4. Western blots analysis of histone proteins in exponential growing cells (WTH3, H3D77A, H3D81A) with indicated antibodies. The relative intensities of H3/GAPDH were quantified using Image J. Data represent the mean ± SE of three independent experiments. (*) *P*<0.05; (**) *P*<0.01.

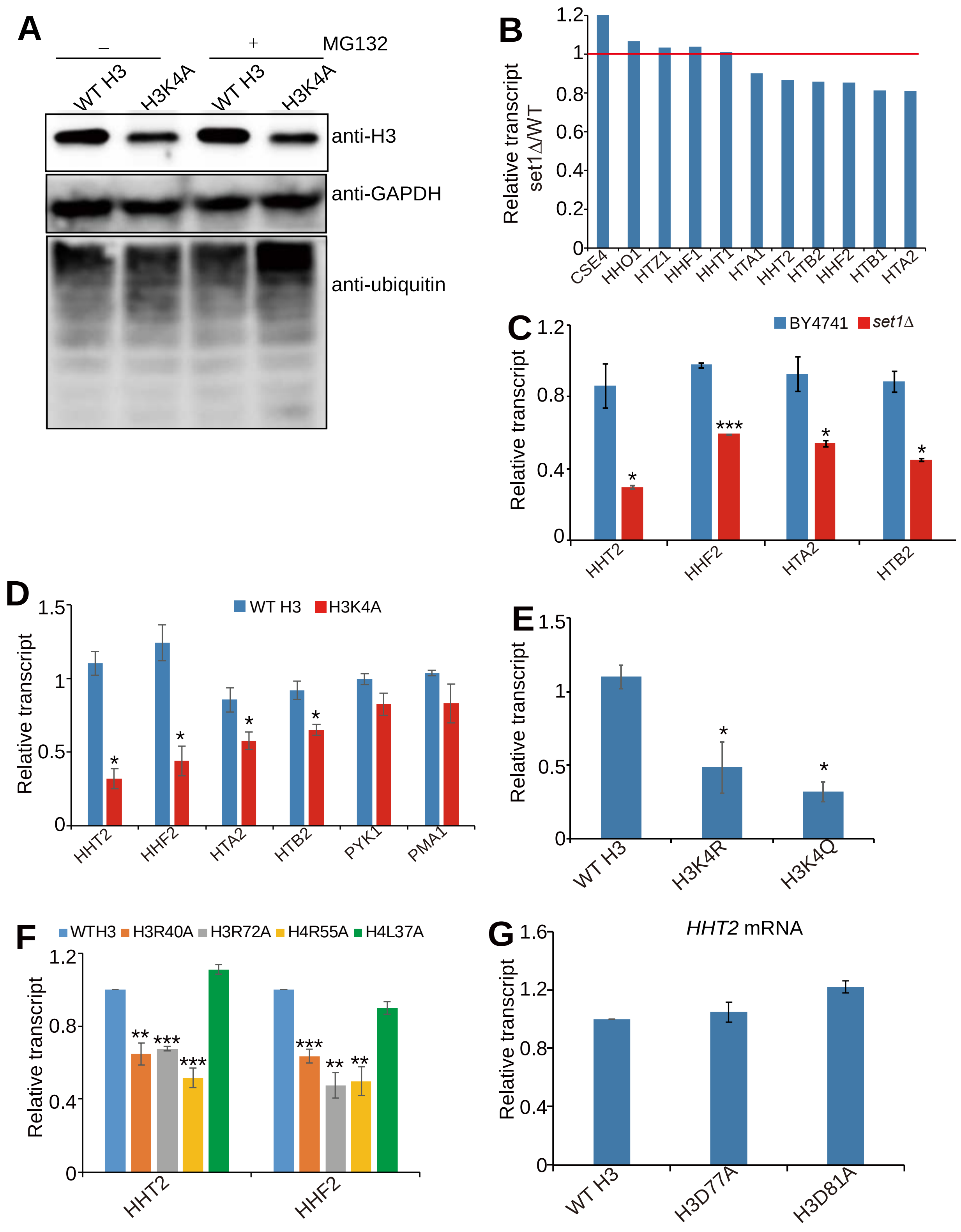
A
–
+
MG132
WT H3
H3K4A
WT H3
H3K4A
anti-H3
anti-GAPDH
anti-ubiquitin
B
Relative transcript set1Δ/WT
1.2
1
0.8
0.6
0.4
0.2
0
CSE4
HHO1
HTZ1
HHF1
HHT1
HTA1
HHT2
HTB2
HHF2
HTB1
HTA2
C
BY4741
set1Δ
Relative transcript
1.2
0.8
0.4
0
*
***
*
*
HHT2
HHF2
HTA2
HTB2
D
WT H3
H3K4A
Relative transcript
1.5
1
0.5
0
*
*
*
*
HHT2
HHF2
HTA2
HTB2
PYK1
PMA1
E
Relative transcript
1.5
1
0.5
0
*
*
WT H3
H3K4R
H3K4Q
F
WTH3
H3R40A
H3R72A
H4R55A
H4L37A
Relative transcript
1.2
0.8
0.4
0
**
***
***
***
**
**
HHT2
HHF2
G
HHT2 mRNA
Relative transcript
1.6
1.2
0.8
0.4
0
WT H3
H3D77A
H3D81A

Figure S5. Set1-catalyzed H3K4me3 positively regulates histone gene transcription. (A) H3K4me3 does not affect histone protein stability. Exponential growing yeast cells (WT H3, H3K4A) were grown in YPD medium in the presence or absence of MG132. The cells were harvested, lysed and analyzed by Western blots with indicated antibodies. (B) Core histone gene expression was reduced in *set1Δ* mutant by RNA-seq. Linker histone (*HHO1*) and histone variants (*CSE4* and *HTZ1*) were not reduced in *set1Δ* mutant. Data were extracted from RNA-seq by Ramakrishnan et al. 2016. (C-D) qRT-PCR analysis of the transcription of *HHT2*, *HHF2*, *HTA2*, *HTB2*, *PYK1* and *PMA1* in WT, *set1Δ,* WT H3 and H3K4A. *PYK1* and *PMA1* were used as negative controls. The RNA levels were normalized to *ACTIN*. Data represent the mean ± SE of three independent experiments. (E) qRT-PCR analysis of the transcription of *HHT2* in WT H3, H3K4R, and H3K4Q cells. (F) qRT-PCR analysis of the transcription of *HHT2* and *HHF2* in WT H3, H3R40A, H3R72A, H4R55A, and H4L37A cells. The transcription of histone genes was significantly reduced in H3R40A, H3R72A and H4R55A mutants but not affected in H4L37A mutant. (G) H3D77A and H3D81A have no significant effect on histone gene transcription as determined by qRT-PCR analysis. Data represent the mean ± SE of three independent experiments. (*) $P<0.05$; (**) $P<0.01$; (***) $P<0.001$.

Supplementary_Fig_S6

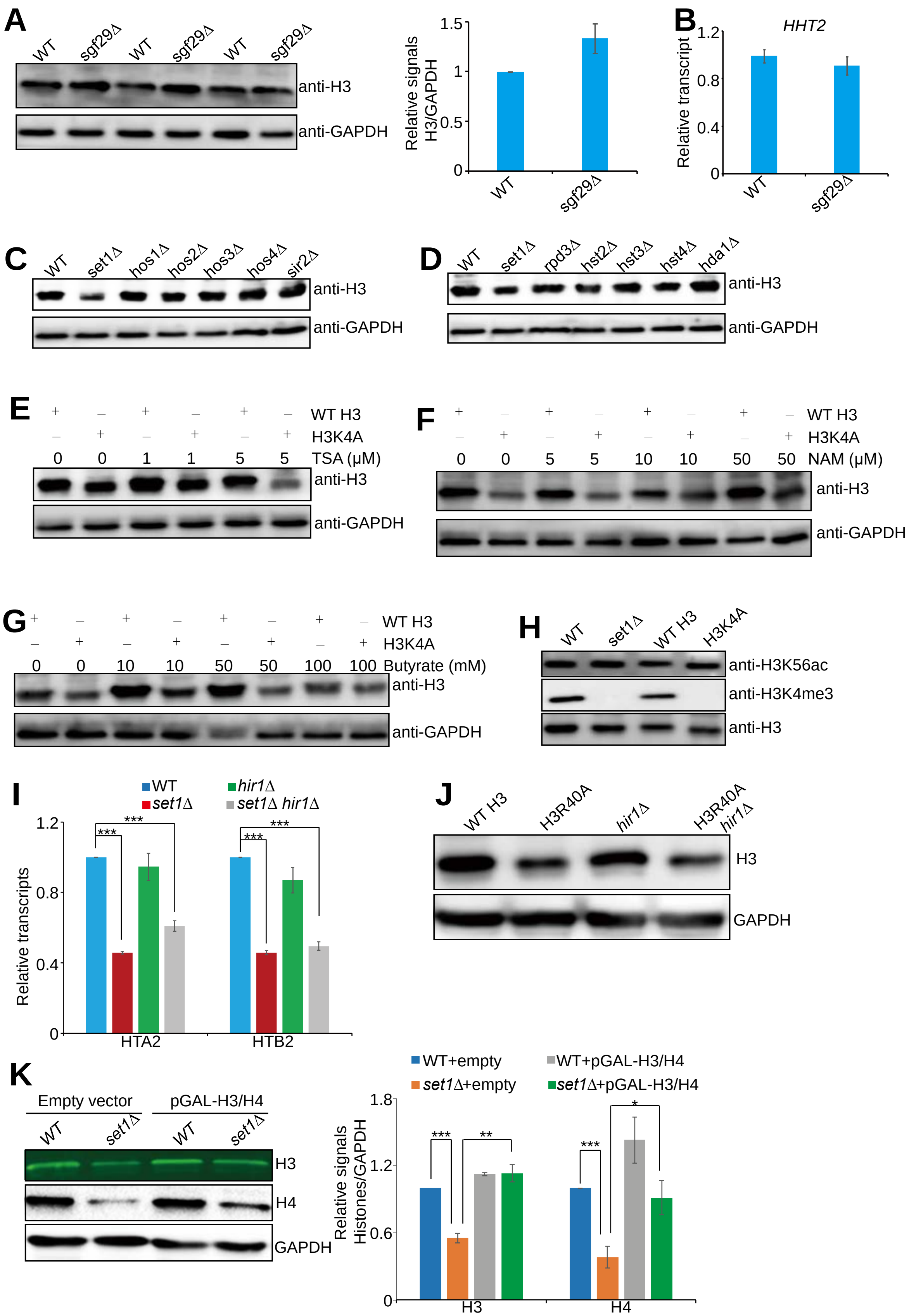

Figure S6. Set1-catalyzed H3K4me3 regulates histone H3 expression not by affecting histone acetylation. (A) Western blots analysis of histone H3 in WT and *sgf29*Δ with indicated antibodies. GAPDH was served as an endogenous control. Right panel: The relative intensities of H3/GAPDH in the left panel were quantified using Image J with standard error (SE) (n=3). (B) Sgf29 has no significant effect on histone gene transcription as determined by qRT-PCR analysis. (C) Western blots analysis of histone H3 in WT, *set1*Δ, *hos1*Δ, *hos2*Δ, *hos3*Δ, *hos4*Δ, and *sir2*Δ. (D) Western blots analysis of histone H3 in WT, *set1*Δ, *rpd3*Δ, *hst2*Δ, *hst3*Δ, *hst4*Δ, and *hda1*Δ. (E) Western blots analysis of histone H3 in WT H3 and H3K4A mutant treated with different concentrations of TSA. TSA cannot rescue the reduced histones in H3K4A mutant. (F) Western blots analysis of histone H3 in WT H3 and H3K4A mutant treated with different concentrations of nicotinamide (NAM) (0, 5, 10, 50 μM). NAM cannot rescue the reduced histones in H3K4A mutant. (G) Western blots analysis of histone H3 in WT H3 and H3K4A mutant treated with different concentrations of sodium butyrate (0, 10, 50, 100 mM). Sodium butyrate cannot rescue the reduced histones in H3K4A mutant. (H) Western blots analysis of histone H3K56ac in exponential growing cells (WT, *set1*Δ, WT H3, H3K4A). (I) qRT-PCR analysis of *HTA2* and *HTB2* transcripts in exponential growing cells (WT, *set1Δ*, *hir1Δ*, *set1Δhir1Δ*). The RNA levels of these genes were normalized to *ACTIN*. Data represent the mean ± SE of three independent experiments. (J) Western blots analysis of histones in exponential growing cells (WT H3, H3R40A, *hir1*Δ, H3R40A *hir1*Δ). Deletion of *HIR1* cannot rescue the reduced histones in H3R40A mutant. (K) Analysis of histone proteins in WT and *set1*Δ cells that were transformed with a vector expressing an extra copy of H3 and H4 under the control of the pGAL1/10 divergent promoters (pGAL-H3/H4). As a control, WT and *set1*Δ cells transformed with empty vector were used. All cells were grown in SC – leucine + 2% galactose for 3 hrs and protein levels were analzyed by Western blots. (*) P<0.05; (**) P<0.01; (***) P<0.001.

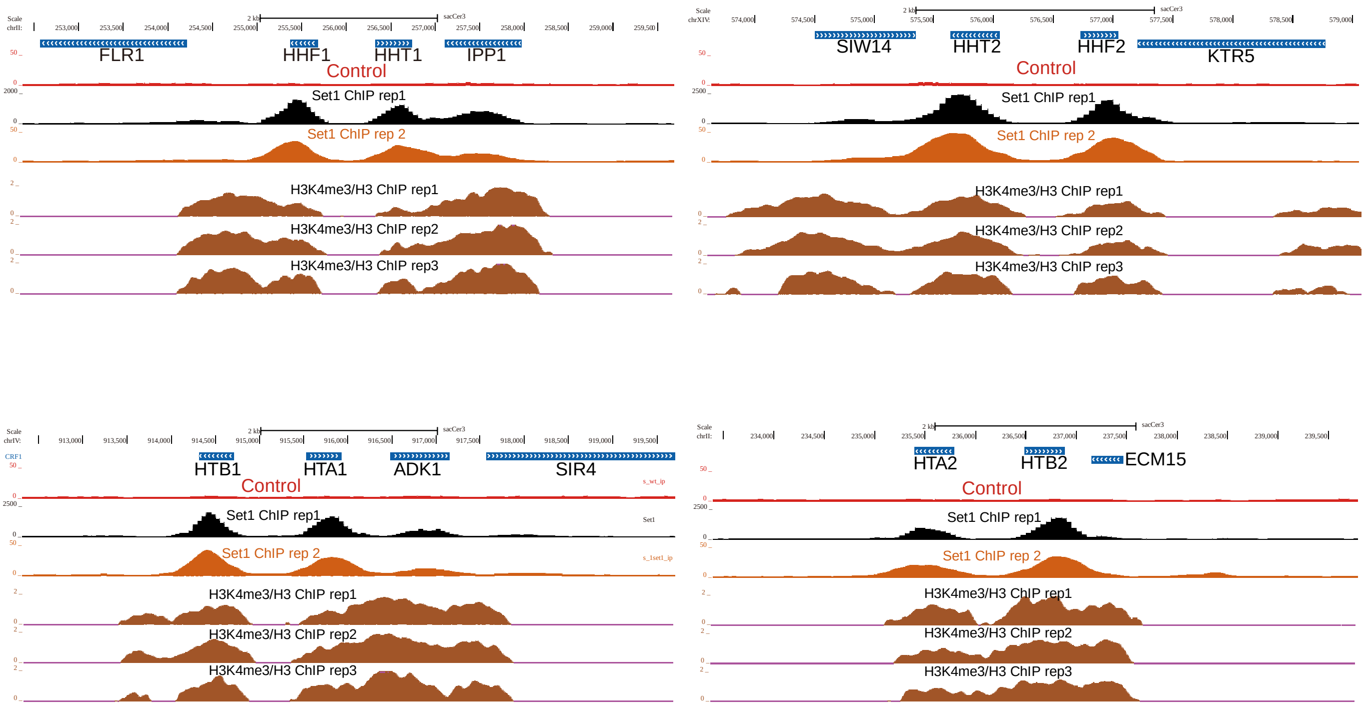

Figure S7. ChIP-seq analysis of the localization of Set1 and H3K4me3/H3 at *HHF1-HHT1*, *HHT2-HHF2*, *HTB1-HTA1*, and *HTA2-HTB2* in exponential growing WT cells. Shown are three biological replicates of ChIP-seq for H3K4me3/H3. Two independent biological replicates of Set1 ChIP-seq data were retrieved from GSE81822 and GSE72972.

Supplementary_Fig_S8

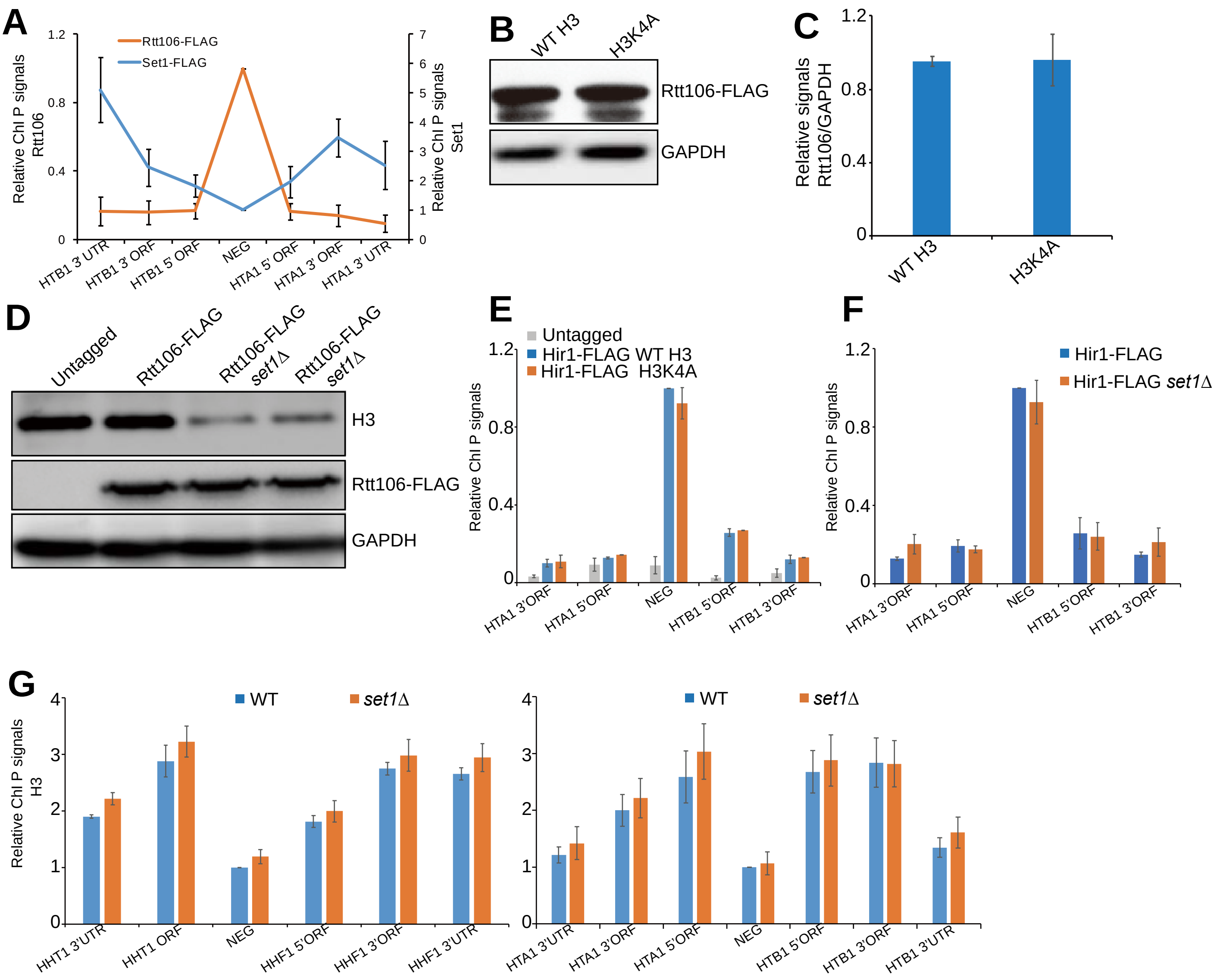

Figure S8. (A) ChIP-qPCR analysis of the binding of Rtt106 and Set1 at *HTB1-HTA1*. The data were presented as the relative ratio of ChIP signal over input signal. (B) Western blots analysis of Rtt106-FLAG levels in WT and H3K4A with anti-FLAG antibody. (C) The relative intensities of Rtt106-FLAG/GAPDH in Figure S8B were quantified using Image J with standard error (SE) (n=3). The protein level of Rtt106 was not significantly changed in H3K4A mutant. (D) Western blots analysis of Rtt106-FLAG levels in WT and *set1*Δ with anti-FLAG antibody. The protein level of Rtt106 was not significantly changed in *set1*Δ mutant. (E-F) ChIP-qPCR analysis of the binding of Hir1 at *HTB1-HTA1* in WT H3, H3K4A (E), WT and *set1Δ* mutant (F). The data were presented as the relative ratio of ChIP signal over input signal. (G) ChIP-qPCR analysis of the occupancy of H3 in *HHT1-HHF1* and *HTA1-HTB1* loci in WT and *set1*Δ mutant. The data were presented as the relative ratio of H3 ChIP signal over input signal. Histone occupancy at these histone gene loci was unaffected by *SET1* deletion. Data represent the mean ± SE of three independent experiments.

Table S1 List of strains used in this study

Name	Parental Strain	Genotype	Source
BY4741	BY4741	*MATa his3Δ1 leu2Δ0 met15Δ0 ura3Δ0*	
set1Δ	BY4741	*MATa his3Δ1 leu2Δ0 met15Δ0 ura3Δ0 set1Δ::KAN*	Open Biosystems
set2Δ	BY4741	*MATa his3Δ1 leu2Δ0 met15Δ0 ura3Δ0 set2Δ::KAN*	Open Biosystems
hir1Δ	BY4741	*MATa his3Δ1 leu2Δ0 met15Δ0 ura3Δ0 hir1Δ::KAN*	Open Biosystems
hir2Δ	BY4741	*MATa his3Δ1 leu2Δ0 met15Δ0 ura3Δ0 hir2Δ::KAN*	Open Biosystems
hir3Δ	BY4741	*MATa his3Δ1 leu2Δ0 met15Δ0 ura3Δ0 hir3Δ::KAN*	Open Biosystems
asf1Δ	BY4741	*MATa his3Δ1 leu2Δ0 met15Δ0 ura3Δ0 asf1Δ::KAN*	Open Biosystems
yta7Δ	BY4741	*MATa his3Δ1 leu2Δ0 met15Δ0 ura3Δ0 yta7Δ::KAN*	Open Biosystems
rtt106Δ	BY4741	*MATa his3Δ1 leu2Δ0 met15Δ0 ura3Δ0 rtt106Δ::KAN*	Open Biosystems
set1Δ rtt106Δ	BY4741	*MATa his3Δ1 leu2Δ0 met15Δ0 ura3Δ0 rtt106Δ::KAN set1Δ::HIS3*	In this study
set1Δ asf1Δ	BY4741	*MATa his3Δ1 leu2Δ0 met15Δ0 ura3Δ0 asf1Δ::KAN set1Δ::HIS3*	In this study
set1Δ yta7Δ	BY4741	*MATa his3Δ1 leu2Δ0 met15Δ0 ura3Δ0 yta7Δ::KAN set1Δ::HIS3*	In this study
set1Δ hir1Δ	BY4741	*MATa his3Δ1 leu2Δ0 met15Δ0 ura3Δ0 hir1Δ::KAN set1Δ::HIS3*	In this study
shg1Δ	BY4741	*MATa his3Δ1 leu2Δ0 met15Δ0 ura3Δ0 shg1Δ::KAN*	Open Biosystems
spp1Δ	BY4741	*MATa his3Δ1 leu2Δ0 met15Δ0 ura3Δ0 spp1Δ::KAN*	Open Biosystems
bre2Δ	BY4741	*MATa his3Δ1 leu2Δ0 met15Δ0 ura3Δ0 bre2Δ::KAN*	Open Biosystems
swd3Δ	BY4741	*MATa his3Δ1 leu2Δ0 met15Δ0 ura3Δ0 swd3Δ::KAN*	Open Biosystems
bre1Δ	BY4741	*MATa his3Δ1 leu2Δ0 met15Δ0 ura3Δ0 bre1Δ::KAN*	Open Biosystems
rad6Δ	BY4741	*MATa his3Δ1 leu2Δ0 met15Δ0 ura3Δ0 rad6Δ::KAN*	Open Biosystems
sgf29Δ	BY4741	*MATa his3Δ1 leu2Δ0 met15Δ0 ura3Δ0 sgf29Δ::KAN*	Open Biosystems
hos1Δ	BY4741	*MATa his3Δ1 leu2Δ0 met15Δ0 ura3Δ0 hos1Δ::KAN*	Open Biosystems
hos2Δ	BY4741	*MATa his3Δ1 leu2Δ0 met15Δ0 ura3Δ0 hos2Δ::KAN*	Open Biosystems
hos3Δ	BY4741	*MATa his3Δ1 leu2Δ0 met15Δ0 ura3Δ0 hos3Δ::KAN*	Open Biosystems
hos4Δ	BY4741	*MATa his3Δ1 leu2Δ0 met15Δ0 ura3Δ0 hos4Δ::KAN*	Open Biosystems
sir2Δ	BY4741	*MATa his3Δ1 leu2Δ0 met15Δ0 ura3Δ0 sir2Δ::KAN*	Open Biosystems
rpd3Δ	BY4741	*MATa his3Δ1 leu2Δ0 met15Δ0 ura3Δ0 rpd3Δ::KAN*	Open Biosystems
hst2Δ	BY4741	*MATa his3Δ1 leu2Δ0 met15Δ0 ura3Δ0 hst2Δ::KAN*	Open Biosystems
hst3Δ	BY4741	*MATa his3Δ1 leu2Δ0 met15Δ0 ura3Δ0 hst3Δ::KAN*	Open Biosystems
hst4Δ	BY4741	*MATa his3Δ1 leu2Δ0 met15Δ0 ura3Δ0 hst4Δ::KAN*	Open Biosystems
hda1Δ	BY4741	*MATa his3Δ1 leu2Δ0 met15Δ0 ura3Δ0 hda1Δ::KAN*	Open Biosystems
ubp8Δ	BY4741	*MATa his3Δ1 leu2Δ0 met15Δ0 ura3Δ0 ubp8Δ::KAN*	Open Biosystems
ubp10Δ	BY4741	*MATa his3Δ1 leu2Δ0 met15Δ0 ura3Δ0 ubp10Δ::KAN*	Open Biosystems
ubp8Δ ubp10Δ	BY4741	*MATa his3Δ1 leu2Δ0 met15Δ0 ura3Δ0 ubp8Δ::KAN ubp10Δ::HIS3*	In this study
Rtt106-FLAG	BY4741	*MATa his3Δ1 leu2Δ0 met15Δ0 ura3Δ0 RTT106-3xFLAG::KAN*	In this study
Rtt106-FLAG *set1Δ*	BY4741	*MATa his3Δ1 leu2Δ0 met15Δ0 ura3Δ0 RTT106-3xFLAG::KAN set1Δ::HIS3*	In this study

HIR1-FLAG	BY4741	*MATa his3Δ1 leu2Δ0 met15Δ0 ura3Δ0 HIR1-3xFLAG::KAN*	In this study
HIR1-FLAG *set1*Δ	BY4741	*MATa his3Δ1 leu2Δ0 met15Δ0 ura3Δ0 HIR1-3xFLAG::KAN set1*Δ*::HIS3*	In this study
YBL574	S288C	*MATa his3Δ200 leu2Δ1 ura3-52, trp1Δ63 lys2-128δ (hht1-hhf1)Δ::LEU2, (hht2-hhf2)Δ::HIS3 Ty912 35-lacZ::his4 pDM18-HHT2-HHF2-TRP1*	In this study
H3K4A (YBL)	S288C	*MATa his3Δ200, leu2Δ1, ura3-52, trp1Δ63, lys2-128δ, (hht1-hhf1)Δ::LEU2, (hht2-hhf2)Δ::HIS3, Ty912 35-lacZ::his4 pDM18-HHT2 (K4A)-HHF2-TRP1*	In this study
H3K4R (YBL)	S288C	*MATa his3Δ200, leu2Δ1, ura3-52, trp1Δ63, lys2-128δ, (hht1-hhf1)Δ::LEU2, (hht2-hhf2)Δ::HIS3, Ty912 35-lacZ::his4 pDM18-HHT2 (K4R)-HHF2-TRP1*	In this study
UCC1369	UCC1369	*MATa ade2::hisG his3Δ200 leu2Δ0 lys2Δ0 met15Δ0 trp1Δ63 ura3Δ0 adh4::URA3-TEL-VIIL AdE2-TEL-VR , hhf2-hht2::MET15 hhf1-hht1::LEU2, pDM18-HHT2-HHF2-TRP1*	(van Leeuwen et al., 2002)
H3K4A (TEL)	UCC1369	*MATa ade2::hisG his3Δ200 leu2Δ0 lys2Δ0 met15Δ0 trp1Δ63 ura3Δ0 adh4::URA3-TEL-VIIL AdE2-TEL-VR , hhf2-hht2::MET15 hhf1-hht1::LEU2, pDM18-HHT2(K4A)-HHF2-TRP1*	In this study
H3K4R (TEL)	UCC1369	*MATa ade2::hisG his3Δ200 leu2Δ0 lys2Δ0 met15Δ0 trp1Δ63 ura3Δ0 adh4::URA3-TEL-VIIL AdE2-TEL-VR , hhf2-hht2::MET15 hhf1-hht1::LEU2, pDM18-HHT2(K4R)-HHF2-TRP1*	In this study
H3K4M (TEL)	UCC1369	*MATa ade2::hisG his3Δ200 leu2Δ0 lys2Δ0 met15Δ0 trp1Δ63 ura3Δ0 adh4::URA3-TEL-VIIL AdE2-TEL-VR , hhf2-hht2::MET15 hhf1-hht1::LEU2, pDM18-HHT2(K4M)-HHF2-TRP1*	In this study
H3R2A (TEL)	UCC1369	*MATa ade2::hisG his3Δ200 leu2Δ0 lys2Δ0 met15Δ0 trp1Δ63 ura3Δ0 adh4::URA3-TEL-VIIL AdE2-TEL-VR , hhf2-hht2::MET15 hhf1-hht1::LEU2, pDM18-HHT2(R2A)-HHF2-TRP1*	In this study
H3T6A (TEL)	UCC1369	*MATa ade2::hisG his3Δ200 leu2Δ0 lys2Δ0 met15Δ0 trp1Δ63 ura3Δ0 adh4::URA3-TEL-VIIL AdE2-TEL-VR , hhf2-hht2::MET15 hhf1-hht1::LEU2, pDM18-HHT2(T6A)-HHF2-TRP1*	In this study
H3K14A (TEL)	UCC1369	*MATa ade2::hisG his3Δ200 leu2Δ0 lys2Δ0 met15Δ0 trp1Δ63 ura3Δ0 adh4::URA3-TEL-VIIL AdE2-TEL-VR , hhf2-hht2::MET15 hhf1-hht1::LEU2, pDM18-HHT2(K14A)-HHF2-TRP1*	In this study
H4K4A-H3K14A (TEL)	UCC1369	*MATa ade2::hisG his3Δ200 leu2Δ0 lys2Δ0 met15Δ0 trp1Δ63 ura3Δ0 adh4::URA3-TEL-VIIL AdE2-TEL-VR , hhf2-hht2::MET15 hhf1-hht1::LEU2, pDM18-HHT2(K4A-K14A)-HHF2-TRP1*	In this study
*set1*Δ (TEL)	UCC1369	*MATa ade2::hisG his3Δ200 leu2Δ0 lys2Δ0 met15Δ0 trp1Δ63 ura3Δ0 adh4::URA3-TEL-VIIL AdE2-TEL-VR , hhf2-hht2::MET15 hhf1-hht1::LEU2, pDM18-HHT2-HHF2-TRP1 set1*Δ*::HIS3*	In this study

*spp1*Δ (TEL)	UCC1369	*MATa ade2::hisG his3Δ200 leu2Δ0 lys2Δ0 met15Δ0 trp1Δ63 ura3Δ0 adh4::URA3-TEL-VIIL AdE2-TEL-VR , hhf2-hht2::MET15 hhf1-hht1::LEU2, pDM18-HHT2-HHF2-TRP1 spp1Δ::HIS3*	In this study
*jhd2*Δ (TEL)	UCC1369	*MATa ade2::hisG his3Δ200 leu2Δ0 lys2Δ0 met15Δ0 trp1Δ63 ura3Δ0 adh4::URA3-TEL-VIIL AdE2-TEL-VR , hhf2-hht2::MET15 hhf1-hht1::LEU2, pDM18-HHT2-HHF2-TRP1 jhd2Δ::HIS3*	In this study
Wide type H3	S288C	*MATa his3Δ200 leu2Δ0 lys2Δ0 trp1Δ63 ura3Δ0 met15Δ0 can1::MFA1pr-HIS3 hht1-hhf1::NatMX4 hht2-hhf2::[HHTS-HHFS]*-URA3*	Open Biosystems
H3K4A	S288C	*MATa his3Δ200 leu2Δ0 lys2Δ0 trp1Δ63 ura3Δ0 met15Δ0 can1::MFA1pr-HIS3 hht1-hhf1::NatMX4 hht2-hhf2::[HHTS(K4A)-HHFS]*-URA3*	Open Biosystems
H3K4Q	S288C	*MATa his3Δ200 leu2Δ0 lys2Δ0 trp1Δ63 ura3Δ0 met15Δ0 can1::MFA1pr-HIS3 hht1-hhf1::NatMX4 hht2-hhf2::[HHTS(K4Q)-HHFS]*-URA3*	Open Biosystems
H3K4R	S288C	*MATa his3Δ200 leu2Δ0 lys2Δ0 trp1Δ63 ura3Δ0 met15Δ0 can1::MFA1pr-HIS3 hht1-hhf1::NatMX4 hht2-hhf2::[HHTS(K4R)-HHFS]*-URA3*	Open Biosystems
H3R2A	S288C	*MATa his3Δ200 leu2Δ0 lys2Δ0 trp1Δ63 ura3Δ0 met15Δ0 can1::MFA1pr-HIS3 hht1-hhf1::NatMX4 hht2-hhf2::[HHTS(R2A)-HHFS]*-URA3*	Open Biosystems
H3T6A	S288C	*MATa his3Δ200 leu2Δ0 lys2Δ0 trp1Δ63 ura3Δ0 met15Δ0 can1::MFA1pr-HIS3 hht1-hhf1::NatMX4 hht2-hhf2::[HHTS(T6A)-HHFS]*-URA3*	Open Biosystems
H3K14A	S288C	*MATa his3Δ200 leu2Δ0 lys2Δ0 trp1Δ63 ura3Δ0 met15Δ0 can1::MFA1pr-HIS3 hht1-hhf1::NatMX4 hht2-hhf2::[HHTS(K14A)-HHFS]*-URA3*	Open Biosystems
H3K14Q	S288C	*MATa his3Δ200 leu2Δ0 lys2Δ0 trp1Δ63 ura3Δ0 met15Δ0 can1::MFA1pr-HIS3 hht1-hhf1::NatMX4 hht2-hhf2::[HHTS(K14Q)-HHFS]*-URA3*	Open Biosystems
H3L37A	S288C	*MATa his3Δ200 leu2Δ0 lys2Δ0 trp1Δ63 ura3Δ0 met15Δ0 can1::MFA1pr-HIS3 hht1-hhf1::NatMX4 hht2-hhf2::[HHTS(L37A)-HHFS]*-URA3*	Open Biosystems
H3R40A	S288C	*MATa his3Δ200 leu2Δ0 lys2Δ0 trp1Δ63 ura3Δ0 met15Δ0 can1::MFA1pr-HIS3 hht1-hhf1::NatMX4 hht2-hhf2::[HHTS(R40A)-HHFS]*-URA3*	Open Biosystems
H3R72A	S288C	*MATa his3Δ200 leu2Δ0 lys2Δ0 trp1Δ63 ura3Δ0 met15Δ0 can1::MFA1pr-HIS3 hht1-hhf1::NatMX4 hht2-hhf2::[HHTS(R72A)-HHFS]*-URA3*	Open Biosystems
H3D77A	S288C	*MATa his3Δ200 leu2Δ0 lys2Δ0 trp1Δ63 ura3Δ0 met15Δ0 can1::MFA1pr-HIS3 hht1-hhf1::NatMX4 hht2-hhf2::[HHTS(D77A)-HHFS]*-URA3*	Open Biosystems
H3D81A	S288C	*MATa his3Δ200 leu2Δ0 lys2Δ0 trp1Δ63 ura3Δ0 met15Δ0 can1::MFA1pr-HIS3 hht1-hhf1::NatMX4 hht2-hhf2::[HHTS(D81A)-HHFS]*-URA3*	Open Biosystems
H4R55A	S288C	*MATa his3Δ200 leu2Δ0 lys2Δ0 trp1Δ63 ura3Δ0 met15Δ0 can1::MFA1pr-HIS3 hht1-hhf1::NatMX4 hht2-hhf2::[HHTS-HHFS(R55A)]*-URA3*	Open Biosystems

H3D77A *hir1*Δ	S288C	*MATa his3Δ200 leu2Δ0 lys2Δ0 trp1Δ63 ura3Δ0 met15Δ0 can1::MFA1pr-HIS3 hht1-hhf1::NatMX4 hht2-hhf2::[HHTS(D77A)-HHFS]*-URA30 hir1Δ::KAN*	In this study
H3D81A *hir1*Δ	S288C	*MATa his3Δ200 leu2Δ0 lys2Δ0 trp1Δ63 ura3Δ0 met15Δ0 can1::MFA1pr-HIS3 hht1-hhf1::NatMX4 hht2-hhf2::[HHTS(D81A)-HHFS]*-URA3 hir1Δ::KAN*	In this study
Rtt106-FLAG WT H3	S288C	*MATa his3Δ200 leu2Δ1 ura3-52, trp1Δ63 lys2-128δ (hht1-hhf1)Δ::LEU2, (hht2-hhf2)Δ::HIS3 Ty912 35-lacZ::his4 pDM18-HHT2-HHF2-TRP1, RTT106-3xFLAG::KAN*	In this study
Rtt106-FLAG H3K4A	S288C	*MATa his3Δ200, leu2Δ1, ura3-52, trp1Δ63, lys2-128δ, (hht1-hhf1)Δ::LEU2, (hht2-hhf2)Δ::HIS3, Ty912 35-lacZ::his4 pDM18-HHT2 (K4A)-HHF2-TRP1, RTT106-3xFLAG::KAN*	In this study
Hir1-FLAG WT H3	S288C	*MATa his3Δ200 leu2Δ1 ura3-52, trp1Δ63 lys2-128δ (hht1-hhf1)Δ::LEU2, (hht2-hhf2)Δ::HIS3 Ty912 35-lacZ::his4 pDM18-HHT2-HHF2-TRP1, HIR1-3xFLAG::KAN*	In this study
Hir1-FLAG H3K4A	S288C	*MATa his3Δ200, leu2Δ1, ura3-52, trp1Δ63, lys2-128δ, (hht1-hhf1)Δ::LEU2, (hht2-hhf2)Δ::HIS3, Ty912 35-lacZ::his4 pDM18-HHT2 (K4A)-HHF2-TRP1, HIR1-3xFLAG::KAN*	In this study
H3K36A	S288C	*MATa his3Δ200 leu2Δ0 lys2Δ0 trp1Δ63 ura3Δ0 met15Δ0 can1::MFA1pr-HIS3 hht1-hhf1::NatMX4 hht2-hhf2::[HHTS(K36A)-HHFS]*-URA3*	Open Biosystems
H3K79A	S288C	*MATa his3Δ200 leu2Δ0 lys2Δ0 trp1Δ63 ura3Δ0 met15Δ0 can1::MFA1pr-HIS3 hht1-hhf1::NatMX4 hht2-hhf2::[HHTS(K79A)-HHFS]*-URA3*	Open Biosystems
Rtt106-FLAG WT H3	S288C	*MATa his3Δ200 leu2Δ1 ura3-52, trp1Δ63 lys2-128δ (hht1-hhf1)Δ::LEU2 (hht2-hhf2)Δ::HIS3 Ty912 35-lacZ::his4 pDM18-HHT2-HHF2-TRP1, RTT106-3xFLAG::KAN*	In this study
Rtt106-FLAG H3K4A	S288C	*MATa his3Δ200, leu2Δ1, ura3-52, trp1Δ63, lys2-128δ, (hht1-hhf1)Δ::LEU2, (hht2-hhf2)Δ::HIS3, Ty912 35-lacZ::his4 pDM18-HHT2 (K4A)-HHF2-TRP1, RTT106-3xFLAG::KAN*	In this study
Hir1-FLAG	BY4741	*MATa his3Δ1 leu2Δ0 met15Δ0 ura3Δ0 HIR1-3xFLAG::KAN*	In this study
Hir1-FLAG *set1*Δ	BY4741	*MATa his3Δ1 leu2Δ0 met15Δ0 ura3Δ0 HIR1-3xFLAG::KAN set1Δ::HIS3*	In this study
HTB1-FLAG WT H3	S288C	*MATa his3Δ200 leu2Δ1 ura3-52, trp1Δ63 lys2-128δ (hht1-hhf1)Δ::LEU2, (hht2-hhf2)Δ::HIS3 Ty912 35-lacZ::his4 pDM18-HHT2-HHF2-TRP1, HTB1-3xFLAG::KAN*	In this study
HTB1-FLAG H3K4A	S288C	*MATa his3Δ200, leu2Δ1, ura3-52, trp1Δ63, lys2-128δ, (hht1-hhf1)Δ::LEU2, (hht2-hhf2)Δ::HIS3, Ty912 35-lacZ::his4 pDM18-HHT2 (K4A)-HHF2-TRP1, HTB1-3xFLAG::KAN*	In this study

HTB1-FLAG	BY4741	*MATa his3Δ1 leu2Δ0 met15Δ0 ura3Δ0 HTB1-3xFLAG::KAN*	In this study
HTB1-FLAG *set1*Δ	BY4741	*MATa his3Δ1 leu2Δ0 met15Δ0 ura3Δ0 HTB1-3xFLAG::KAN set1*Δ*::HIS3*	In this study
JKM139		*MATa hmlΔ hmrΔ ade1 lys5 leu2-3,112 trp1::hisG ura3-52 ho ade3::GAL-HO (hta2-htb2)Δ::KAN*	Provided by Dr. Xuefeng Chen
HTB1K123R		*hoΔ hml::ADE1 MATEa hmr::ADE1 ade1-110 leu2,3-112 lys5 trp1::hisG ura3-52 ade3::GAL-HO (hta2-htb2)Δ::KAN HTB1(K123R)-TRP1*	Provided by Dr. Xuefeng Chen
Eno2-FLAG	BY4741	*MATa his3Δ1 leu2Δ0 met15Δ0 ura3Δ0 ENO2-3xFLAG::KAN*	In this study
BY4741 pGAL-H3/H4	BY4741	*MATa his3Δ1 leu2Δ0 met15Δ0 ura3Δ0 pESC-LEU2-pGAL1/10-HHT2-HHF2*	In this study
*set1*Δ pGAL-H3/H4	BY4741	*MATa his3Δ1 leu2Δ0 met15Δ0 ura3Δ0 set1*Δ*::KAN pESC-LEU2-pGAL1/10-HHT2-HHF2*	In this study

Table S2 List of oligonucleotides used in this study

Gene name	Sequence
ChIP	
PHO11	GACAAAATCGGAACTCAAACGG
	TCTTTCACCGTGTCTACCAAC
SOR2	TCATGAATCAAGCGGACAGG
	GTTCAATAGCAACACGGTCAC
YCR102C	GTTGCCTACAAATCACCCAATG
	AGGTTCAAGCCCAAGTTATAGG
THI12	ACCAATCCTTCCGATGTCAC
	AAAGAGGCAACAGAGGTCAC
YDL241W	CATGGCTTCACAAATGCGAG
	TTCTGTACCTTTCCCTTGCAG
1kb	GTTATGTTAGAGATAACTGTGAG
	GCTTGTTAACTCTCCGACAG
2.5kb	GCAATGAATCTTCGGTGCTTGG
	CCATACCAATATCAACTTCACGG
5kb	CCCCGCCTTTGAAGATTGTCCC
	CGAGACCCACTTGTATTCTTAGTGC
7.5kb	CCTCTATAGGACCTGTCTCATGG
	GGAAGTCTACACTAATAGCTATGCG
30.5K	CAACTCACCCAATCCGTCTAG
	TCCAACTCAGCAATAGCGTC
64.8K	AAGGGTTTTGGGTTTGTTCAC
	TCCAATTGAGAGTCACGTTCC
Chr VI-R-eu	GGATTGACGGGTAACCCTAAAAGG
	GTTGTCATGGCCAATGACCACGAT
HTA1-HTB1 A	CGAAACTTCAGAGCATTGGC
	GGGTTCAATCTCCAAGGCAT
HTA1-HTB1 B	CCAGGTGAATTGGCTAAGCA
	GCATTCCCTCTATGAGACCA
HTA1-HTB1 C	AGAGAAGCAAGGCTAGAAAGGA
	GGAAATACCAGTGTCAGGGTG
HTA1-HTB1 D	CCACAAATAAACCATACACAC
	GGAAATACCAGTGTCAGGGT
HTA1-HTB1 E	CGCTCAATGTCGCCCGAAAG
	TGCCCCTTTCTTACCAATCGTTA
HTA1-HTB1 F	ATAGTTAACGACCCAACCGCGT
	ACGGGCGTTTCTTCAACAACGA
HTA1-HTB1 G	CGCCTCACTGTGCGAAGCTATT
	TCCACTGGCTGGCTTCGTGA
HTA1-HTB1 H	ACGGATTTGGTTATTTCTCAGTGAA

	ATTCCAAGACAGCAGTCAAGTAGAC
HTA1-HTB1 I	GGTTCTGGTGCTCCAGTCTAC
	TCTTCTTGTTATCCCTAGCAGCAT
HTA1-HTB1 J	CAAAGAAGTCTGCCAAGGCT
	AGCAGTTTAGTTCCTTCCGC
HTA1-HTB1 K	AGGTTCATTGGGCACTGTTG
	ACAGTTCTCCGTGACAGGAT
HHT1-HHF1 A	ATCTGAGAGCAGGAAGAGCA
	GTGTGTCAGCATCAGAGGTT
HHT1-HHF1 B	TCATCAGAGACTCTGTTACC
	GTTACCGTTTTCTTAGAATTAG
HHT1-HHF1 C	CCGCGAATACGGTGGTAAAT
	TGGCACCACCTTTACCTAGA
HHT1-HHF1 D	CCTCTACCGGACATATTTTACT
	TACTTTAGCAAATGCCCGCG
HHT1-HHF1 E	ATTTACCACCGTATTCGCGG
	AGGTGCAGAGCAAGGAAATG
HHT1-HHF1 F	CTCACATTTCCTTGCTCTGCACC
	CAAGGAGCCGTGGAGGGTACC
HHT1-HHF1 G	GCAATTAGCTTCTAAGGCTGCCAG
	GCAGCCAAGTTGGTATCTTCAA
HHT1-HHF1 H	AATCTTCTGCCATCGGTGCC
	CTAAAACTGATGACAATCAAC
HHT1-HHF1 I	GCCTTGTAGGAGGCAAGATT
	CGTATGCGGCTTCAAGTTGT
HHT2-HHF2 A	GCCGTTGAGTGCTTCGACGT
	AACGCGGCCTACGACGAGGG
HHT2-HHF2 B	GGTTCTACCTTGTCTCTTCAAAGC
	GCTAGAAGAGGTGGTGTCAAGCG
HHT2-HHF2 C	TGGTGTCAAGCGTATTTCTGG
	ACAGTCTTTCTCTTGGCGTG
HHT2-HHF2 D	AAATGACCAACTCCCATCCG
	TTTGTTCTGGTCTGGTCTGC
HHT2-HHF2 E	GCCCCAAGAAAACAATTAGCC
	CTCTCAAGGCAACAGTACCTG
HHT2-HHF2 F	GATTGGTCAGAGAAATCGCTCAA
	TCTTAGTCTTCTGGCCAATTTGAT
HHT2-HHF2 G	GCTTTTAACGACATGAGGAGGG
	CGAGATGTATGACGATGACGAAA
qRT-PCR	
HHT2	GCCCCAAGAAAACAATTAGCC
	CTCTCAAGGCAACAGTACCTG

HHF2	TGGTGTCAAGCGTATTTCTGG
	ACAGTCTTTCTCTTGGCGTG
HTA1	GGTTCTGGTGCTCCAGTCTAC
	TCTTCTTGTTATCCCTAGCAGCAT
HTA2	AGCTGGTTTAACATTCCCAGTT
	GCAGTTAGATAGACTGGAGCAC
HTB2	GATTGATCTTACCTGGTGAATTGGCTAAA
	GGCTTGAGTAGAGGAGGAGTAT
ACTIN	CTGTCGAGAGATTTCTCTTTTACC
	GCCCCTATTTATTCCAATAATATCG
PHO11	GACAAAATCGGAACTCAAACGG
	TCTTTCACCGTGTCTACCAAC
SOR2	TCATGAATCAAGCGGACAGG
	GTTCAATAGCAACACGGTCAC
YCR102C	GTTGCCTACAAATCACCCAATG
	AGGTTCAAGCCCAAGTTATAGG
THI12	ACCAATCCTTCCGATGTCAC
	AAAGAGGCAACAGAGGTCAC
YDL241W	CATGGCTTCACAAATGCGAG
	TTCTGTACCTTTCCCTTGCAG
TUBULIN	TCT TGG TGG TGG TAC TGG TT
	TGG ATT TCT TAC CGT ATT CAG CG
TPI1	AGAAGCCACAAGTCACTGTC
	ACCCACTTAGCACCAACATC
PYK1	CCCAATCCCACCAAACCAC
	TTCTACCAGCGGAGATGACCTT
ACS2	TGATTCCAGAAGCGGTCATTG
	GAGAACCCAGCAAAGACAAC
KRE1	CTGTGTCCTCGTCTGTATCTTC
	GAGGTTCTGTGAAGTCTGTGG
PAB1	AAGGGTTTTGGGTTTGTTCAC
	TCCAATTGAGAGTCACGTTCC
BMH1	CAACTCACCCAATCCGTCTAG
	TCCAACTCAGCAATAGCGTC

Table S3 List of reagents or resources used in this study

REAGENT or RESOURCE	Source	Identifier
**Antibodies**		
Anti-Histone H3 (124-135)	Abcam	ab1791
Anti-Histone H3	Abclonal	A2352
Anti-Histone H4	Abcam	Ab10158
Anti-H3K4 monomethylation	Millipore	07-436
Anti-H3K4 dimethylation	Abcam	ab32356
Anti-GAPDH	proteintech	10494-1-AP
Anti-Actin	proteintech	20536-1-AP
Anti-trimethyl-Histone H3 (Lys4) Antibody	Millipore	07-473
Anti-H3K4 trimethylation	Abclonal	A2357
Anti-H3K36 trimethylation	Abclonal	A2366
Anti-H3K14 acetylation	Abclonal	A7254
Anti-H3K56 acetylation	Abclonal	A7256
Anti-Ubiquityl-Histone H2B (Lys120) (D11) XP Rabbit mAb	Cell signaling	5546
Anti-Ubiquitin	Cell signaling	3936S
Anti-FLAG M2	Sigma-Aldrich	F1804-1MG
Anti-Pgk1	Molecular Probe	A-6457
Anti-Rpb3	Neoclone	WP012
**Chemicals, recombinant Proteins**		
α factor	ZYMO RESEARCH	Y1001
Micrococcal Nuclease	Worthington	R3P14588
Anti-FLAG M2 Affinity Gel	Sigma-Aldrich	A2220-5ML
Recombinant histone H3K4me3	Active motif	31278
WT Octamers	Gift from Dr. Bing Li	
H3K4me3 Octamers	Gift from Dr. Bing Li	

References

van Leeuwen, F., Gafken, P. R., and Gottschling, D. E. (2002). Dot1p modulates silencing in yeast by methylation of the nucleosome core. Cell *109*, 745-756.
